# Sodium danshensu modulates skeletal muscle fiber type formation and metabolism by inhibiting pyruvate kinase M1

**DOI:** 10.3389/fphar.2024.1467620

**Published:** 2024-10-22

**Authors:** Yunxia Zhang, Xiaoxiao Wu, Ruoqi Li, Mengru Sui, Guoyin Li, Shuhua Fan, Mingsheng Yang, Qiuping Liu, Xiaomeng Liu, Changjing Wu, Lili Li

**Affiliations:** ^1^ Institute of Translational Medicine, College of Life Science and Agronomy, Zhoukou Normal University, Zhoukou, Henan, China; ^2^ Dancheng Green Agriculture Observation and Research Station of Henan Province, Zhoukou Normal University, Zhoukou, China; ^3^ College of Public Health, Xinxiang Medical University, Xinxiang, Henan, China

**Keywords:** muscle fiber, muscle metabolism, sodium danshensu, MyHCs, pyruvate kinase

## Abstract

Sodium Danshensu (SDSS) is extracted from *Salvia miltiorrhiza* and has many pharmacological effects. However, little is known about its effects on muscle fiber formation and metabolism. Here, we aimed to investigated the role and molecular mechanisms of SDSS in modulating the formation of skeletal muscle fiber. C2C12 cells were incubated in differentiation medium with or without SDSS for 4 days. C57BL/6 mice were orally administered SDSS by gavage once a day for 8 weeks. Grip strength, treadmill, muscle weight, western blotting, qPCR, immunofluorescence staining and H&E staining were performed. SDSS target proteins were searched through drug affinity responsive target stability (DARTS) and mass spectrometry analysis. Furthermore, molecular docking was carried out for Pyruvate kinase M1 (PKM1). The effect of PKM1 on myosin heavy chain (*MyHCs*) gene expression was verified by knockdown of PKM1 experiment. SDSS induced oxidative muscle fiber-related gene expression, and inhibited glycolytic fiber-related gene expression in C2C12 cells. Muscle mass, the percentage of slow oxidative fibers, succinic dehydrogenase activity, muscle endurance, glucose tolerance, and the expression of the *MyHC1* and *MyHC2a* genes increased while *MyHC2b* expression, lactate dehydrogenase activity, and the percentage of glycolytic muscle fibers decreased in SDSS-treated mice. Mechanistically, SDSS bound to the pyruvate kinase PKM1 and significantly repressed its activity. PKM1 inhibited *MyH*C1 and *MyHC2*a expression but promoted *MyHC2b* expression. SDSS also significantly attenuated the effects of PKM1 on muscle fiber-related gene expression in C2C12 cells. Our findings indicate that SDSS promotes muscle fiber transformation from the glycolytic type to the oxidative type by inhibiting PKM1 activity, which provide a new idea for treating muscle atrophy, muscle metabolism diseases and improving animal meat production.

## 1 Introduction

Skeletal muscle is composed of different types of muscle fiber depending on myosin heavy chain (MyHC) isomers. In adult mammals, the main muscle fiber types are slow oxidative, fast oxidative, fast glycolytic, and oxidative-glycolytic muscle fibers (Schiaffino and Reggiani, 2011), of which the molecular markers are MyHC1, MyHC2a, MyHC2b, and MyHC2x, respectively (da Costa et al., 2007). Oxidative fibers contain more myoglobin and mitochondria, which have a higher oxidative capacity, and confer endurance (Ozawa et al., 2000; Westerblad et al., 2010). Muscle fiber type is thought to be an important factor influencing on muscle metabolism, physical exercise capacity, and the development of muscle disease. People with a high proportion of fast glycolytic fibers are at higher risk for obesity and related metabolic diseases (Ceaser and Hunter, 2015). However, the proportion of slow oxidative muscle fibers are inversely related to fatness (Wade et al., 1990). Previous studies have reported an association between a higher proportion of oxidative muscle fibers and greater insulin sensitivity of skeletal muscle (Megeney et al., 1993; Snow and Thompson, 2009). Skeletal muscles with a high proportion of slow oxidative fibers are less prone to atrophy than those largely made up of fast glycolytic fibers (Wang and Pessina, 2013). In animals raised for meat production, the proportion of slow oxidative muscles correlates positively with meat quality, and the proportion of fast glycolytic muscles correlates with the level of pale, soft, and exudative meat (Ryu et al., 2008). Therefore, elucidating the molecular mechanisms that regulate the formation of muscle fiber type is particularly important for improving both the treatment of muscle diseases and meat quality traits.

In recent years, many studies have shown that the formation and transformation of muscle fiber types can be regulated by natural compounds, including resveratrol (Jiang et al., 2019), quercetin (Chen et al., 2021), lauric acid (Wang et al., 2018), ferulic acid (Chen et al., 2019), and oleanolic acid (Liu et al., 2024). Danshen (*Salvia miltiorrhiza*), belonging to the Labiatae family, is an important traditional Chinese herbal plant with a long history as a medicine and health food (Leung et al., 2010; Zhang et al., 2011). Danshensu (DSS, also known as salvianic acid A) is extracted from Danshen (Bao et al., 2018). Its demonstrated effects include relaxation of the coronary arteries (Li et al., 2018) and protection against myocardial ischemia-reperfusion injury (Wu et al., 2007; Zhang et al., 2021). DSS has been used to treat myocardial infarction (Gao et al., 2022) and has been shown to inhibit cancer cell migration and invasion (Kumar et al., 2020). However, its effects on muscle function have not been investigated. DSS is a water-soluble phenolic acid component of Danshen, but it is inherently unstable (Yang and Shen, 2022; Zhang et al., 2019). As the sodium salt of DSS, sodium Danshensu (SDSS) has high stability and high rates of absorption and utilization (Wang et al., 2022; Meng et al., 2019). In the study we examined the effects and mechanisms of SDSS on the conversion of skeletal muscle fiber type and muscle metabolism. Our findings indicated that SDSS can promote the transformation of muscle fiber from the glycolytic muscle fiber to the oxidative muscle fiber both *in vitro* and *in vivo*. Mechanistically, SDSS regulated the expression of *MyHCs* genes by binding PKM1. These results provide a new idea for treating metabolic diseases and improving animal meat production.

## 2 Material and method

### 2.1 Cell culture and treatment

C2C12 myoblasts (ATCC) were cultured in 10% fetal bovine serum (FBS) (Gibco, Australia) in high-glucose Dulbecco’s modified Eagle’s medium (DMEM, Waltham, MA, United States) at 37°C and 5% CO_2_. The cells were plated in DMEM with 10% FBS (Gibco, Australia). When the cells reached 80% confluence, they were induced to differentiate by adding 2% horse serum (Gibco, ham, MA, United States) for 4 days. In the SDSS treatments, SDSS was dissolved in PBS and PBS was used as the control. The cells were treated with 20 μM SDSS during C2C12 myoblasts differentiation. SDSS was purchased from Aladdin (Aladdin, Shanghai, China, CAS: 67920–52–9), The purity of SDSS is greater than 98.5%.

### 2.2 Mice and treatments

A total of 30 Four-week-old male C57BL/6J mice were acquired from the Model Animal Research Center of Nanjing University (Nanjing, China). Three or four mice were kept per cage under a 12 h light/dark cycle and a temperature of 23°C ± 2°C. Unrestricted access to water and a chow diet that contained 10% kcal as fat was provided. After 7 days of acclimation, the mice were randomly assigned to three groups with ten replicates. The mice were orally administered 0 mg/kg, 5 mg/kg or 10 mg/kg SDSS for 8 weeks. SDSS was dissolved in saline and saline was used as the control. Mice were euthanized by cervical dislocation, and TA, Gas and Qua muscles of mice were collected and weighed, separately. Data were normalized to the body weight (mg/g). All animal testing was conducted under a protocol approved by the Health Sciences Animal Welfare Committee of Zhoukou Normal University (Permit Number ZKNU-2024009, Permit Date 5 March 2022).

### 2.3 Measurement of grip strength and exercise endurance

The mice were orally administered 0 mg/kg, 5 mg/kg or 10 mg/kg SDSS for 8 weeks. A grid strength meter (LE902, Panlab, Spain) was used to test mouse grab strength as follows. The mouse was gently placed on the grip test network, with the lever of the grip tester and the body of the mouse oriented in the same direction. After the test value had been set to zero, the mouse was quickly pulled back and the maximum grip strength of the mouse was recorded. The test was conducted five times, and the maximal strength was determined. The treadmill-running test was performed on an XP-PT-10B animal treadmill (Shanghai Xinruan, China) as follows. An awake mouse was placed on the treadmill set to an initial speed of 10 m/min for 3 min, after which the speed was increased to 30 m/min to induce fatigue (Meng et al., 2020). When the mouse was no longer able to stay on the treadmill, the total running distance up to that point was recorded.

### 2.4 Assessment of glucose homeostasis and oxygen consumption

The mice were orally administered 0 mg/kg, 5 mg/kg or 10 mg/kg SDSS for 8 weeks. The mice were fasted for 16 h before they were tested with a glucose tolerance test (GTT). Tail blood glucose was measured with an Accu-Chek glucometer (Roche Diagnostics Corp) at 0, 15, 30, 45, 60, 90 and 120 min after an intraperitoneally administered injection of glucose at 1.5 g/kg. A TSE lab master system was used to detect O_2_ consumption (TSE Systems, Germany). Before the measurements, the mice were allowed to acclimate for 24 h. The volume of O_2_ consumed within 24 h was determined. For all experiments, the mice were housed at 25°C in a 12 h light/12 h dark cycle, with free access to food and water.

### 2.5 RNA isolation and quantitative real-time PCR (qPCR)

Total RNA was isolated from cells or muscle tissues using Trizol reagent (Invitrogen, California, United States). The RNA concentration was measured on a NanoDrop 2000 (Thermo, Wilmington, UAS). Agarose gel electrophoresis was used to determine the quality of the RNA. Total RNA (1 µg) was reverse transcribed with PrimeScript RTreagent with gDNA Eraser (Takara). A LightCycler 96 (Roche, Basel, Switzerland) system was used for qPCR. The primers are listed in Supplementary Table S1. Relative gene expression was analyzed using the Ct (2^−ΔΔCT^) method.

### 2.6 Western blotting

Cells or tissues were lysed in RIPA buffer (Servicebio, Wuhan, China) containing protease and phosphatase inhibitors according to the manufacturer’s instructions. Protein lysates were heated at 95 °C for 5 min in 5 × sodium dodecyl sulfate (SDS) sample buffer and were separated by 10% SDS-PAGE (30 μg each lane); then, the gel was transferred to polyvinylidene fluoride (PVDF) membranes (Millipore, United States) using a Mini Trans-Blot Cell system (Bio-Rad, United States). The membrane was blocked with 5% non-fat milk for 1.5 h. The primary antibodies were incubated overnight at 4°C. The membranes were washed and incubated with secondary antibodies for 1 h at room temperature. The membranes were visualized by ECL (Bio-Rad, United States). The primary antibodies targeted MyHC1 (Abcam, United States, Cambridge, ab11083, 1:5000), MyHC2a (Santa Cruz, United States, sc-53095, 1:200), MyHC2b (ABclonal, Wuhan, China, A15293, 1:500), MyHC2x (Abcam, United States, ab127539, 1:500), PKM1 (Proteintech, Wuhan, China, 15821-1-AP, 1:3,000) and β-actin (Boster, China, BM0627, 1:1,000). The secondary ones were IgG-HRP-conjugated antibodies. Bands on the membranes were visualized by ECL (Bio-Rad, United States).

### 2.7 H&E and immunofluorescence staining

Fixed tissues were sectioned after being embedded in paraffin. Sections with 5 μm thickness were stained with H&E then images were acquired by microscope (Bai et al., 2023). The mean area of muscle fibers from each animal was determined using ImageJ software. Immunofluorescence staining of cells and tissues was performed according to a previously published method (Bai et al., 2023). The primary antibodies used in immunofluorescence staining were MyHC2a (Santa Cruz, United States, sc-53095, 1:50), MyHC2b (ABclonal, Wuhan, China, A15293, 1:50), and MyHC1 (Abcam, United States, Cambridge, ab11083, 1:500). The secondary antibodies were anti-mouse-FITC (Beyotime, Shanghai, China, A0562; 1:300), anti-rabbit-FITC (Beyotime, Shanghai, China, A0568; 1:300), and anti-rabbit-Cy3 (Beyotime, Shanghai, China, A0516; 1:500). After the secondary antibody step, the cell nuclei were stained with DAPI during incubation in the dark. Images were captured via confocal laser scanning microscopy (LSM800; Zeiss, Oberkochen, Germany). The protein expression level of MyHC2a, MyHC2b and MyHC1 was determined by ratio of the number of nuclei within MyHC isoforms positive myotubes to the total number of nuclei.

### 2.8 Measurement of lactate content and enzyme activity in muscles

0.1 g muscle tissue were added in 900 μL saline, and use a frozen tissue grinder (KZ-III-FP, Servicebio, Wuhan, China) to prepare a 10% homogenate. The working conditions of frozen tissue grinder are 70 Hz for 45 s, with an interval of 15 s, repeated 4 times. The protein concentration was quantified using a BCA protein assay kit (Beyotime, Shanghai, China, P0010). The amount of lactate, lactate dehydrogenase (LDH) activity, and succinate dehydrogenase (SDH) activity were measured using commercial kits (A019–2–1, A020–1, A022–1–1, Nanjing Jiancheng Bioengineering Institute, China) according to the manufacturer’s instructions.

### 2.9 Drug affinity responsive target stability (DARTS)

C2C12 myoblasts were differentiated for 4 days and then lysed in M-PER (Thermo Scientific, Waltham, MA, United States) containing protease and phosphatase inhibitors. The lysates were diluted with TNC buffer (50 mM Tris-HCl pH 8.0, 50 mM NaCl, 10 mM CaCl_2_) and the protein concentration was quantified using a BCA protein assay kit (Beyotime, Shanghai, China, P0010). The lysates were incubated under the control condition or with 20 µM SDSS for 60 min at 4°C (Yang et al., 2017; Lomenick et al., 2009). Then they were quickly warmed to 37°C and proteolyzed by adding 1 µg thermolysin to 15 µg lysate. After 10 min, the reaction was stopped by adding 0.5 M EDTA (pH 8.0) to each sample at a 1:10 ratio. Proteins in the samples were separated by SDS–PAGE and visualized by Coomassie staining. Then mass spectrometry or western blotting analysis was conducted.

### 2.10 Mass spectrometry (MS) analysis

Gel bands were cut out and prepared for MS analysis as follows. The proteins were reduced by incubation with 25 mM DTT for 40 min at 37°C and then digested with sequencing-grade trypsin at 37°C overnight. The supernatant was desalinated on a C18 solid-phase cartridge and freeze-dried (Wu et al., 2023). The dried peptides were loaded onto a triple TOF 5600+LC-MS system (AB SCIEX, United States). Protein identification and annotation were performed using ProteinPilot (version 4.5, SCIEX, California, United States).

### 2.11 Molecular docking

To investigate the interaction and binding activities of the SDSS with the selected protein, molecular docking analysis was subsequently carried out. The SDSS complex was analyzed on the Yinfo Cloud Platform (http://cloud.yinfotek.com/). The structure of SDSS was built with energy minimization using MMFF94 forcefield. The PKM1 crystal structure was extracted from the Protein Data Bank (https://www.rcsb.org/; PDB code: 2G50) (Williams et al., 2006) and then adjusted by removing water, ions, and the original ligands. A grid box (26 × 26 × 26 Å) was prepared using AutoGrid, with the center of the box set at the coordinates x = −6.217, y = 29.935, and z = −10.689 with respect to the Arg105 residue. Flexible docking was conducted using default parameters (Trott and Olson, 2010). The nine top-ranked ligand–receptor conformations sorted by the calculated free energy of binding were retained. Discovery Studio Visualizer v21.1.0.20298 (Accelrys, San Diego, United States) was used to visualize the best conformation, and the interaction modes were analyzed.

### 2.12 Plasmids, siRNA synthesis, and cell transfection

For *PKM1* overexpression plasmids, the full-length sequence of PKM1, containing *BamH* I and *Xba* I sites, was inserted into plasmid pcDNA3.1 by Tsingke Biotechnology (Wuhan, China). A *PKM1* siRNA sequence was synthesized by GenePharma (Shanghai, China). The *PKM1* siRNA oligonucleotide sequences were as follows: sense: GUU​CCA​CCG​UCU​GCU​GUU​UTT; antisense: AAA​CAG​CAG​ACG​GUG​GAA​CTT. C2C12 myoblasts seeded in 12-well plates were transfected with 2 µg or 100 pmol siRNA using Lipofectamine 2000 (Invitrogen, California, United States) according to the manufacturer’s instruction. Then, the cells were induced to differentiate by 2% horse serum for 4 days.

### 2.13 Pyruvate kinase (PK) activity measurements

C2C12 cells seeded in 12-well plates were differentiated for 4 days and treated with different concentrations of SDSS, digested with 0.25% trypsin, and then washed with PBS. A BCA protein assay kit (Beyotime, Shanghai, China, P0010) was used to measure the protein concentration; PK activities were measured using a commercial kit (Solarbio, Beijing, China, BC0540) according to the manufacturer’s protocol. The absorbance at 340 nm was determined and used to calculate PK activity. The enzyme activity was calculated as follows: PK activity (U/mg protein) = 2,680 × △A/protein content, △A is absorbance change within 2 min. PK activity in the control group was normalized to 1.

### 2.14 Statistical analysis

Differences between groups were analyzed using Student’s t-test or one-way ANOVA. The results are presented as the mean ± standard deviation. A P-value <0.05 was considered to indicate statistical significance.

## 3 Results

### 3.1 SDSS upregulates the expression of oxidative muscle fiber-related genes

**FIGURE 1:**
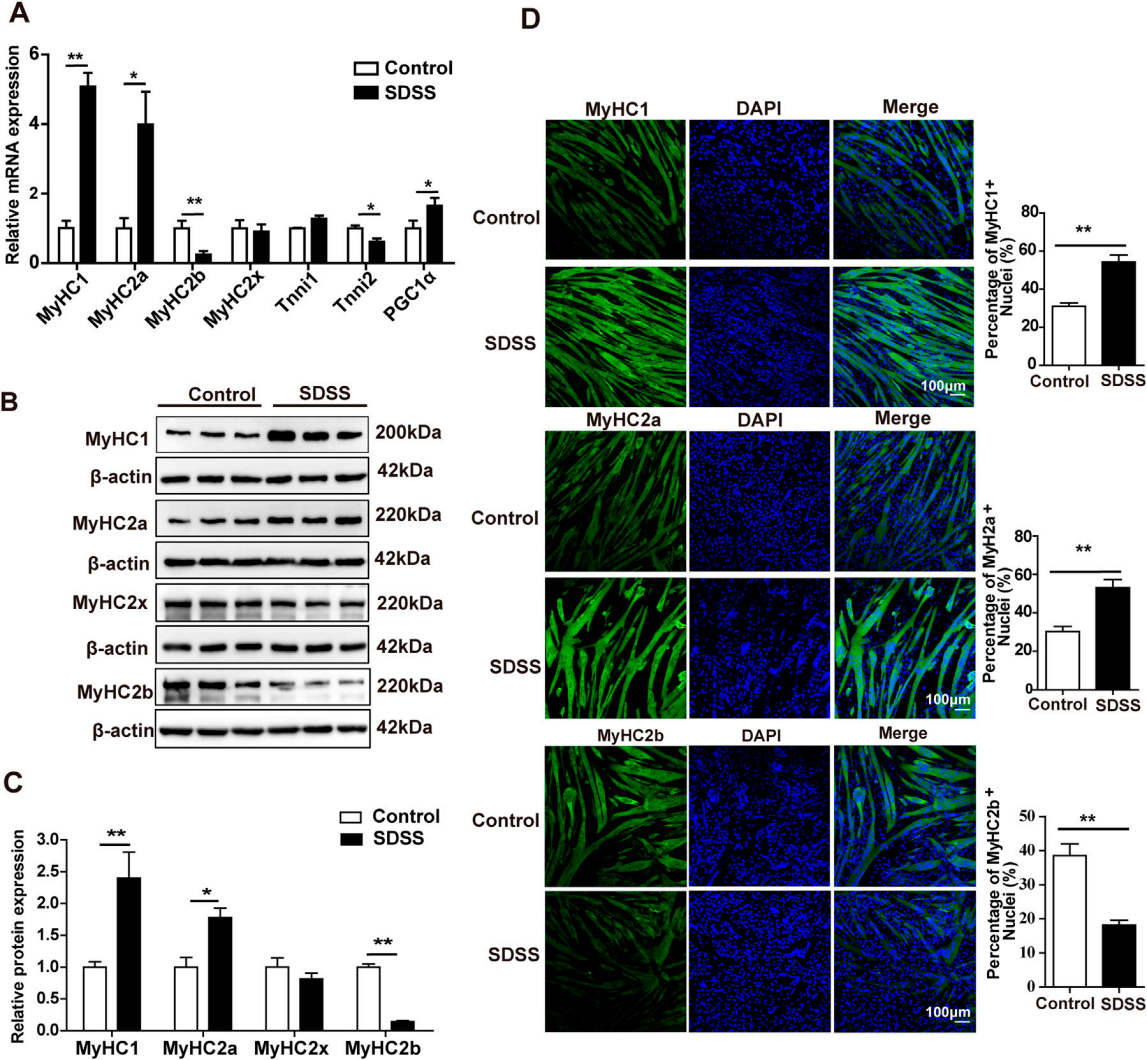
SDSS affects the expression of genes involved in the determination of muscle fiber type in C2C12 myoblasts. **(A)** qPCR analysis of the mRNA levels of genes related to muscle fiber type. **(B)** Western blotting analysis of MyHC1, MyHC2a, MyHC2x, and MyHC2b protein expression. **(C)** Average relative protein levels were normalized to the amount of β-actin. **(D)** Immunofluorescence staining and quantification of MyHC1, MyHC2a, and MyHC2b. C2C12 myotubes were stained with antibodies (green; including anti-MyHC1, anti-MyHC2a, and anti-MyHC2b) and DAPI (blue). Scale bar, 100 μm. Data are presented as the mean ± SD. *p < 0.05, **p < 0.01.

We first studied the cytotoxicity of SDSS on C2C12 myoblasts. Compared with 0 µM SDSS (PBS), no difference in cell viability was observed in C2C12 myoblasts treated for 24 h with less than or equal to 80 µM SDSS, loss of cell viability was seen in concentrations exceeding 80 µM SDSS treatment (Supplementary Figure S1). To explore the effects of natural products on the muscle development and oxidative muscle fiber formation, we screened some natural products, which had a significant impact on the MyHC1 gene expression in C2C12 cells. Our preliminary experimental results showed that SDSS had a significant impact on the MyHC1 gene expression. To determine the optimal concentration of SDSS, C2C12 cells were treated with 0, 10, 20, 40, 60 and 80 µM drug. At 20 μM, SDSS significantly upregulated the expression of the *MyHC1* gene and downregulated that of the *MyHC2b* gene (Supplementary Figure S2). Therefore, 20 μM SDSS was used in subsequent experiments. The qPCR results showed that 20 μM SDSS significantly increased the expression of *MyHC1*, *MyHC2a*, and *PGCα* mRNA and decreased the expression of *MyHC2b* and *Tnni2* mRNA but had no effect on the expression of *MyHC2x* and *Tnni1* mRNA compared to the control group (Figure 1A). Western blotting and immunofluorescence staining indicated that 20 μM SDSS also significantly increased the expression of MyHC1 and MyHC2a proteins and reduced the expression of MyHC2b protein (Figures 1B–D), while the expression of MyHC2x protein did not significantly change (Figures 1B, C). These findings show that SDSS upregulates the expression of oxidative muscle fiber-related genes, and downregulates the expression of glycolytic muscle fiber-related genes.

### 3.2 SDSS increases muscle mass and enhances muscle endurance

**FIGURE 2:**
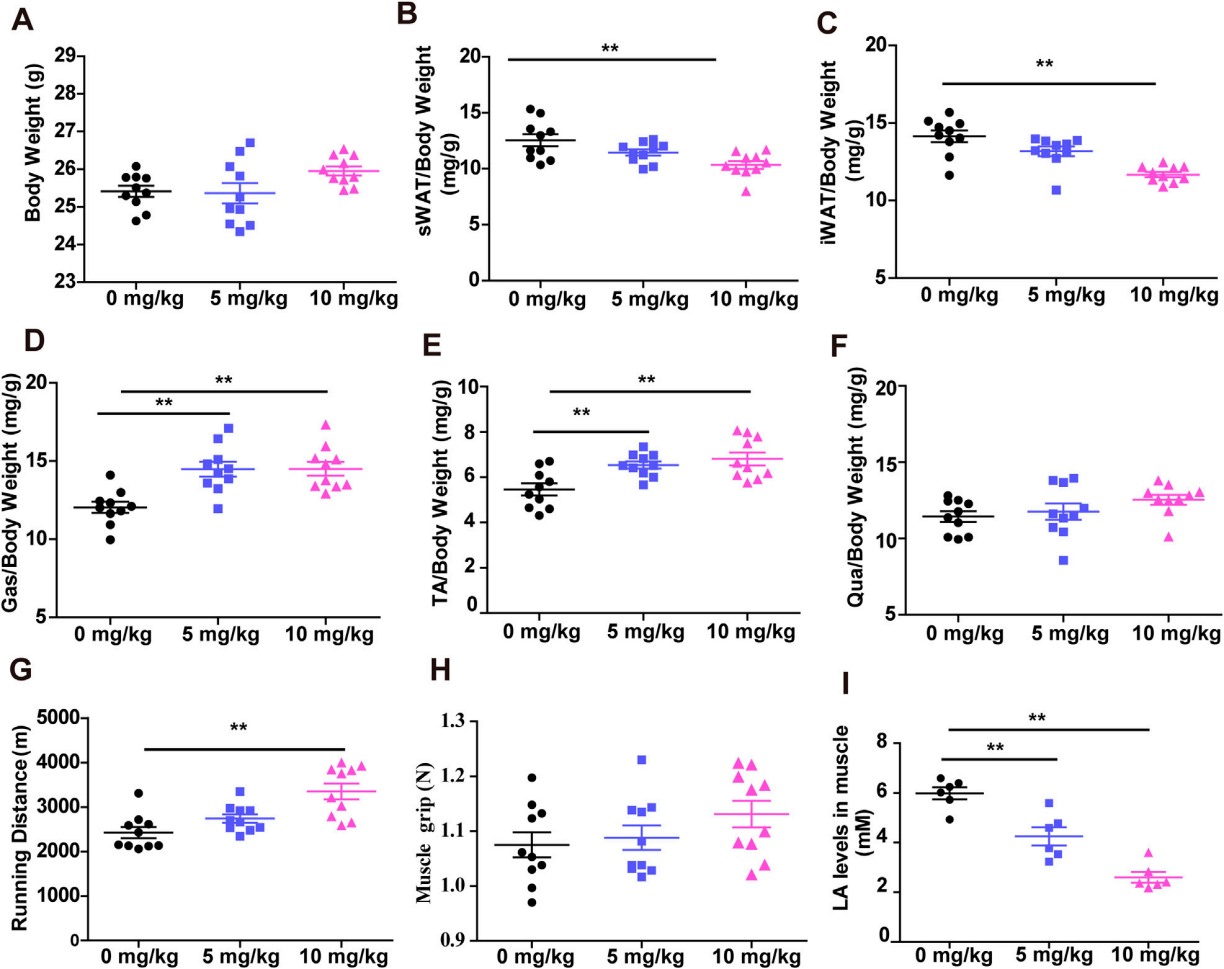
SDSS increases muscle mass and endurance. Mice were orally administered SDSS (5 or 10 mg/kg/day) by gavage once a day for 8 weeks. **(A)** Body weight of mice treated with normal saline or SDSS (5 or 10 mg/kg/day). **(B–F)** The ratio of subcutaneous white adipose tissue (sWAT) **(B)**, inguinal white adipose tissue (iWAT) **(C)**, and gastrocnemius (Gas) **(D)**, tibialis anterior (TA) **(E)**, and quadriceps (Qua) **(F)** muscle tissue to body weight after treatment (n = 10). **(G)** Grip strength of SDSS-treated mice (n = 10). **(H)** Running distances of SDSS-treated mice (n = 10). **(I)** Lactic acid content in SDSS-treated mice (n = 6). **p < 0.01.

The effects of SDSS on skeletal muscle were examined in mice treated via gavage with SDSS at a dose of 5 mg/kg or 10 mg/kg per day for 8 weeks; the control group was given normal saline. There were no significant changes in body weight between the SDSS groups (5 mg/kg and 10 mg/kg) and the control group (Figure 2A), but there were obvious changes in white adipose tissue (WAT) and muscle mass (Figures 2B–E). Mice given 10 mg/kg SDSS showed a reduction in subcutaneous WAT (sWAT) and inguinal WAT (iWAT) weight, while no change were seen in mice treated with 5 mg/kg SDSS (Figures 2B, C). The two groups of SDSS-treated mice (5 mg/kg and 10 mg/kg) showed an increase in gastrocnemius (Gas) and tibialis anterior (TA) muscle weight (Figures 2D, E), but quadriceps (Qua) mass did not change significantly (Figure 2F). Both the grip strength and the exercise capacity of the mice were measured to investigate the effects of SDSS on muscle function. Compared to controls, the 10 mg/kg SDSS group had a longer running distances before exhaustion (Figure 2G), but there were no significant differences in grip strength among the three groups (Figure 2H). Both two groups of SDSS-treated mice had reduced lactic acid levels in muscle and in serum (Figure 2I; Supplementary Figure S3), indicating that SDSS relieves muscle fatigue. In summary, our results suggest that SDSS treatment increases muscle mass, prevents muscle fatigue, and enhances muscle endurance.

### 3.3 SDSS promotes fast glycolytic -to-slow oxidative muscle fiber type transformation

**FIGURE 3:**
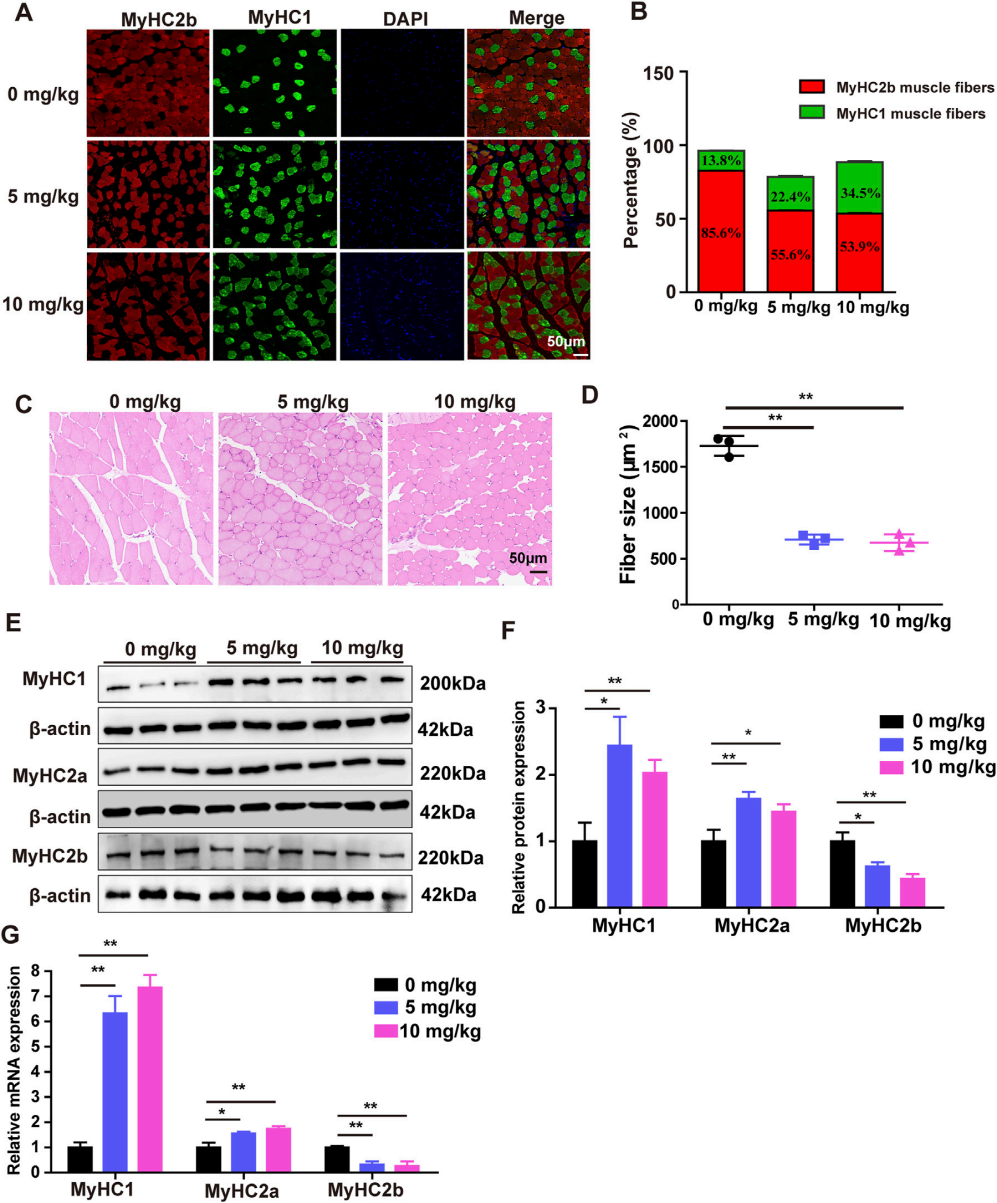
SDSS affects the expression of genes related to muscle fiber type in mice. Mice were administered SDSS (0, 5 or 10 mg/kg/day) by gavage once a day for 8 weeks. **(A)** Immunofluorescence analysis of MyHC2b and MyHC1 muscle fiber types in the Gas muscles of SDSS-treated mice. MyHC2b muscle fibers are indicated in red, MyHC1 muscle fibers are green, and DAPI-stained nuclei are blue. Scale bars, 50 μm. **(B)** The levels of MyHC1 and MyHC2b protein expression in the mice shown in **(A)** were determined based on the ratio of the number of nuclei within MyHC-isoform-positive myotubes to the total number of nuclei. **(C)** H&E staining for the Gas muscles of mice, Scale bars, 50 μm. **(D)** Quantification in three independent experiments indicated that in muscles of SDSS treatment mice significantly decreases the mean cross-sectional areas of individual myofibers. **(E)** Western blotting of proteins encoded by genes involved in muscle fiber type in the Gas muscles of SDSS-treated mice. **(F)** Average relative protein levels were normalized to the amount of β-actin. **(G)** qPCR analysis of mRNA expression of genes related to muscle fiber type in the Gas muscles of SDSS-treated mice. The data are presented as the mean ± SD. *p < 0.05, **p < 0.01.

To determine whether the muscle fiber type conversion following SDSS treatment, Immunofluorescence staining was performed on Gas muscle to determine the effect of SDSS treatment on muscle fiber type transformation. There was an increase in the percentage of slow oxidative fibers (MyHC1 muscle fibers) in response to 5 mg/kg and 10 mg/kg SDSS, with the higher dose also inducing a decrease in the percentage of fast glycolytic fibers (MyHC2b muscle fibers) (Figures 3A, B). H&E staining showed that the mean cross-sectional areas of individual myofibers of the Gas muscles were significtantly decreased by 5 mg/kg and 10 mg/kg SDSS treatment (Figures 3C, D). Western blotting results showed significant increases in the expression of MyHC1 and MyHC2a proteins, and a significant decrease in the expression of MyHC2b protein in both treated groups vs. control mice (Figures 3E, F). The immunofluorescence and western blotting results were confirmed by qPCR (Figure 3G). These results suggest that SDSS promotes muscle fiber type conversion from fast glycolytic muscle to oxidative muscle.

### 3.4 SDSS enhances the oxidative capacity of skeletal muscle *in vivo*

**FIGURE 4:**
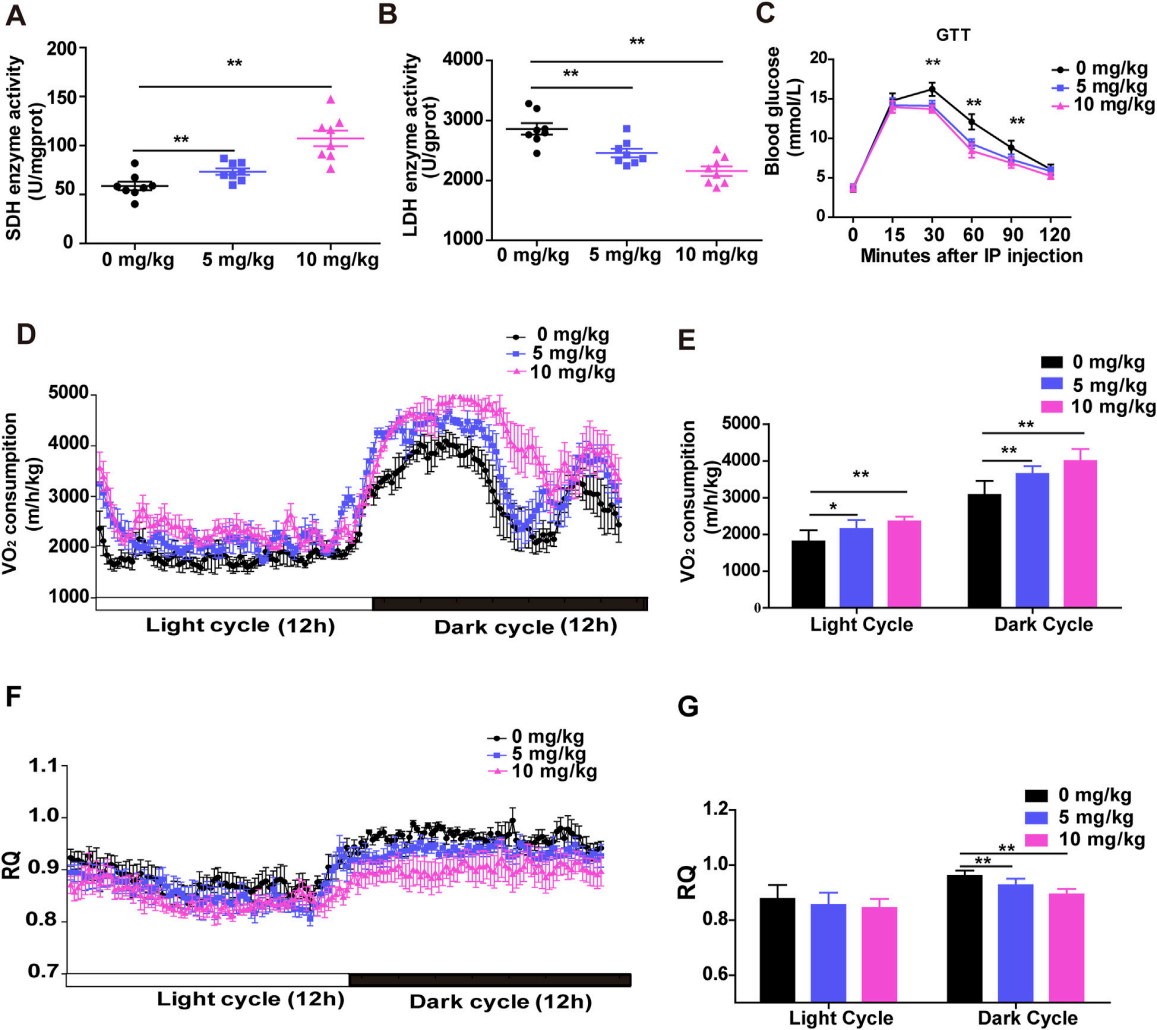
SDSS enhances the oxidative capacity of skeletal muscle *in vivo*. Male C57BL/6J mice were administered SDSS (0, 5 or 10 mg/kg/day) by gavage once a day for 8 weeks. SDH **(A)** and LDH **(B)** activities in Gas muscles (n = 8 per group). **(C)** GTT results of control and SDSS-treated mice injected after an overnight fast with 1.5 g glucose per kg (n = 8 per group). **(D)** O_2_ consumption in control and SDSS-treated mice during a 24 h period (n = 6). **(E)** Average O_2_ consumption in each group. **(F)** The respiration quotient (RQ) of control and SDSS-treated mice (n = 6). **(G)** Average RQ for each group. The data are presented as the mean ± SD of independent experiments. *p < 0.05, **p < 0.01.

Changes in skeletal muscle fiber type lead to changes in metabolic function. Distinct muscle fiber types are closely related to metabolic activity. Oxidative muscle fibers are rich in mitochondria, myoglobin, have high aerobic metabolic enzyme activity and oxidative capacity, and require more oxygen (Meng et al., 2013). As expected, a significant increase in succinate dehydrogenase (SDH) activity and a significant reduction in lactate dehydrogenase (LDH) activity were determined in the Gas muscles of both treated groups compared to the control group (Figures 4A, B). Next, we performed a glucose tolerance test (GTT) to investigate the effect of SDSS on glucose homeostasis. Mice treated with 5 mg/kg or 10 mg/kg SDSS showed significantly faster clearance of glucose from the circulation compared to control mice (Figure 4C), indicating that mice treated with 5 mg/kg or10 mg/kg SDSS had higher glucose tolerance. To validate the enhanced oxidative capacity in the mice from the SDSS-treatment group, we used the metabolic chamber analysis to measure energy influx and consumption in the whole body of both the control and SDSS-treatment group mice. Mice in the two SDSS groups had significantly higher overall O_2_ consumption during both the 12 h dark cycle and the 12 h light cycle (Figures 4D, E) and a decreased respiration quotient (RQ) only during the 12 h dark cycle (Figures 4F, G). These results indicate that SDSS increases energy expenditure during the active phase and that the increases correlate with a higher proportion of oxidative fibers.

### 3.5 SDSS modulates skeletal muscle fiber type transformation by targeting PKM1

**FIGURE 5:**
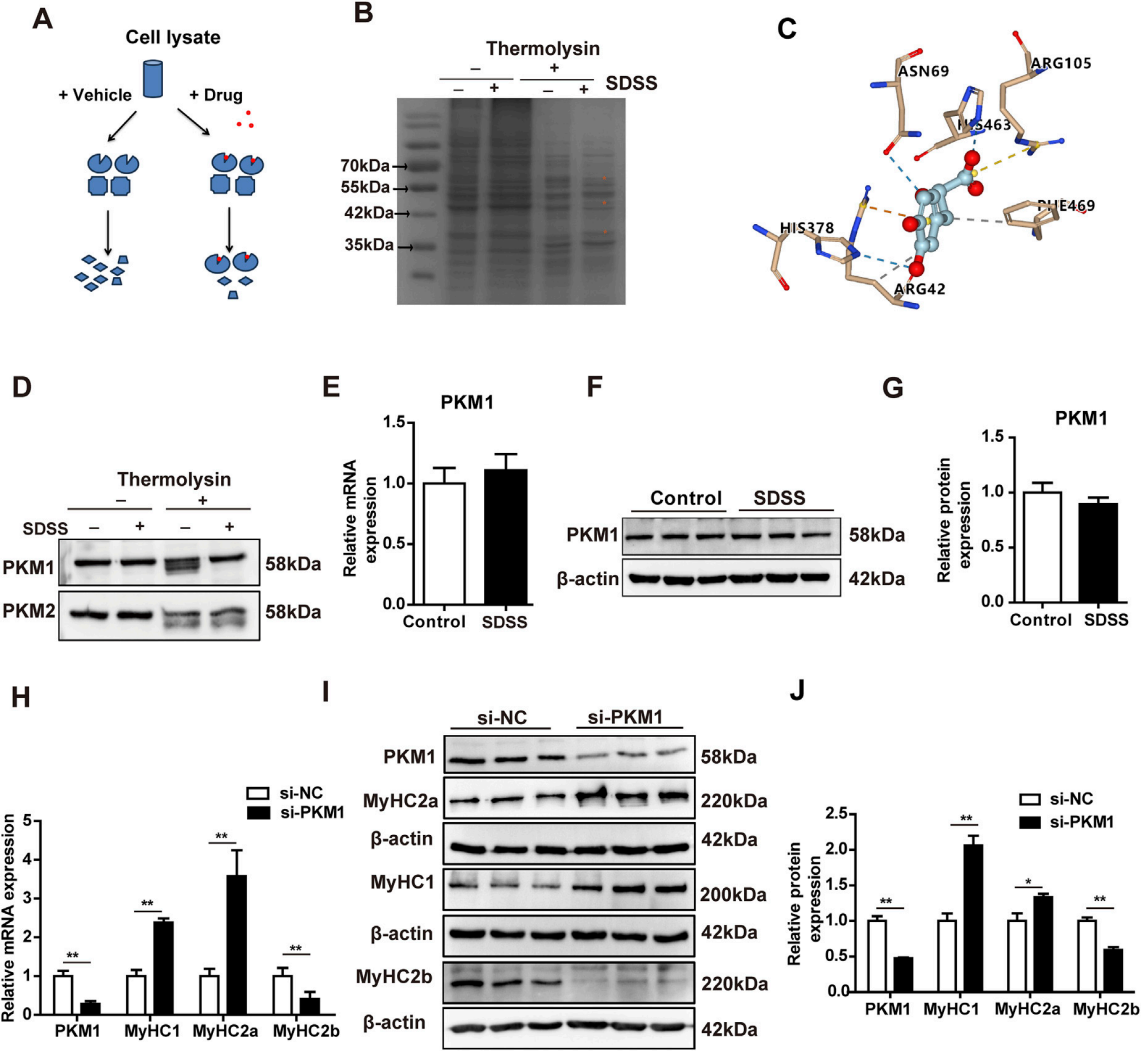
SDSS target identification and its regulation of the expression of genes related to muscle fiber type. **(A)** Scheme of the DARTS method for drug target identification. **(B)** SDSS bound to its targets as determined by DARTS. C2C12 lysates were incubated (or not) with 20 µM SDSS and then subjected to thermolysin digestion for 60 min. The digested products were resolved via SDS-PAGE and detected by Coomassie blue-staining. *Proteins protected from thermolysin proteolysis. **(C)** Predicted dock conformation in a stick model of the allosteric binding pocket, showing the interactions (dotted line: yellow for salt bridge, blue for hydrogen bonding, orange for π–cation, and gray for hydrophobic interaction) between bound SDSS and protein residues. **(D)** Identification of PKM1 as a target of SDSS by DARTS and western blotting. C2C12 lysates were incubated (or not) with 20 μM SDSS and then subjected to thermolysin digestion for 60 min. The digested products were detected via western blotting. **(E)** qPCR analysis of the mRNA levels of PKM1 genes expression in differentiated C2C12 myoblasts after SDSS treatment. **(F)** Western blotting results of PKM1 protein expression in differentiated C2C12 myoblasts after SDSS treatment. **(G)** PKM1 protein levels were normalized to the amount of β-actin. **(H)** qPCR analysis of the mRNA levels of genes related to muscle fiber type in differentiated C2C12 myoblasts after transfection with NC or si-PKM1 RNA. **(I)** Western blotting results of muscle fiber type related protein expression in differentiated C2C12 myoblasts after transfection with NC or si-PKM1 RNA. **(J)** Average relative protein levels were normalized to the amount of β-actin. The data are presented as the mean ± SD. *p < 0.05, **p < 0.01.

To investigate the molecular mechanism of SDSS regulation of muscle fiber type conversion, Drug affinity responsive target stability (DARTS) and mass spectrometry (MS) was performed to screen for potential SDSS-binding proteins. The basic strategy of DARTS is shown in Figure 5A. Drug binding can stabilize target proteins, e.g., in a specific conformation or by masking protease recognition sites, thereby reducing protease sensitivity of the target protein (Lomenick et al., 2009). DARTS results revealed protected bands of about 55 kDa and 42 kDa in the protease-treated samples incubated with SDSS compared to the same samples without SDSS treatment (Figure 5B). Then cut these two protected bands for MS analysis. MS detected 74 protected proteins, with PKM1 present in high abundance in the SDSS-treated samples (Supplementary Table S2). Previous reports also have shown that PKM1 regulates glucose catabolism (Morita et al., 2018). To better understand the capacity and mechanism of SDSS binding of PKM1, SDSS interaction modes with the enzyme were investigated using Autodock Vina software. SDSS efficiently inserted into the allosteric binding groove of PKM1 and interacted with residues of the enzyme (Supplementary Figure S4). Arg105 formed a salt bridge with the carboxyl of SDSS, which was further coordinated via hydrogen bonding with Asn69, His378, and His463. Both π–cation and hydrophobic interactions between Arg42 and the benzene ring of SDSS were identified, with Phe469 also involved in hydrophobic interactions (Figure 5C). To confirm PKM1 as a target protein of SDSS, we performed DARTS and western blot analyses using cell lysates from C2C12 cells. Western blotting results showed that after thermolysin treatment, the PKM1 protein level in SDSS-treated lysates was significantly increased compared with the vehicle PBS-treated group (Figure 5D), while PKM2 protein was degraded without protection (Figure 5D). To verify whether SDSS affected the expression of PKM1 gene, qPCR and western blotting were used to detect *PKM1* gene expression in C2C12 cells treated with 20 µM SDSS. However, there was no significant change in the expression of *PKM1* between control and SDSS treatment group (Figures 5E–G). To verify the effect of PKM1 on the formation of muscle fiber type, siRNA-mediated PKM1 interference (si-PKM1) were performed in C2C12 cells. The qPCR and western blotting results showed that knockdown of PKM1 in C2C12 cells increased the expression of *MyHC1* and *MyHC2a* and decreased the expression of *MyHC2b* (Figures 5H–J). These results imply that PKM1 promotes *MyHC2b* expression and fast glycolytic muscle fibers formation and represses *MyHC1* and *MyHC2a* expression and oxidative muscle fibers formation.

### 3.6 SDSS modulates skeletal muscle fiber-related genes expression by repressing PKM1 activity

**FIGURE 6:**
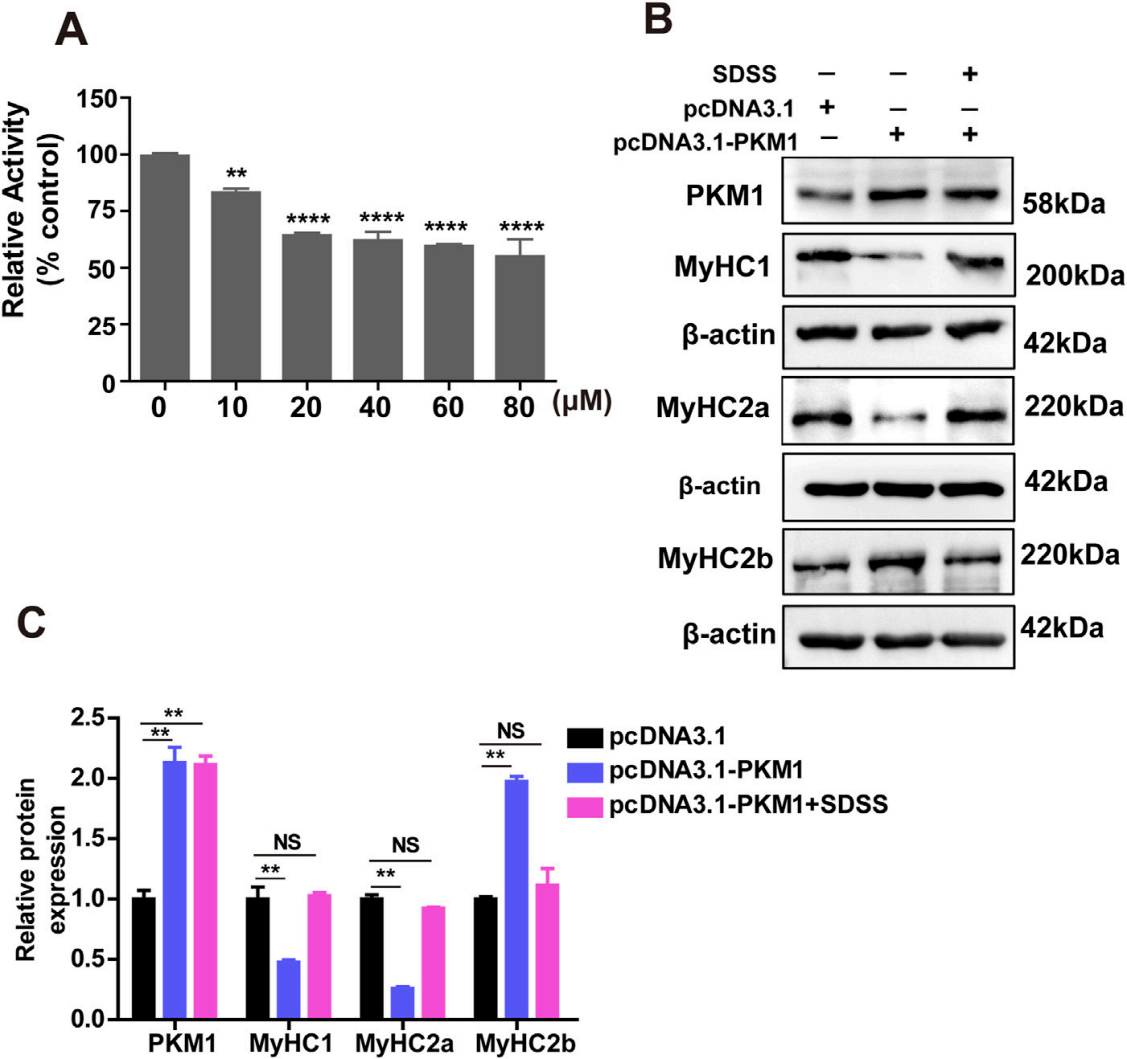
SDSS regulates the expression of genes related to muscle fiber type by inhibiting PKM1 activity. **(A)** Effect of SDSS on PK activity in C2C12 myoblasts differentiated for 4 days. **(B)** Western blotting showing that SDSS reduces the effect of overexpressed PKM1 on the expression of genes related to muscle fiber type. **(C)** Average relative protein levels normalized to the amount of β-actin. The data are presented as the mean ± SD. **p < 0.01, ****p < 0.0001. NS, no significance between the two groups.

To evaluate the mechanism by which SDSS influences the formation of muscle fiber type via PKM1, the effect of SDSS on PKM activity was determined in C2C12 cells. Compared to the control group, PKM activity was significantly repressed by SDSS (Figure 6A). The concentration of SDSS was between 1 and 20 μM, PKM activity decreased as the SDSS concentration increased. However, exceeding 20 µM SDSS treatment, the decrease of PK activity was not proportional to SDSS concentration (Figure 6A). To further investigate whether SDSS modulated the expression of *MyHCs* genes by inhibiting PKM1, we overexpressed *PKM1* gene in C2C12 cells treated with 20 µM SDSS. The expression of *MyHCs* genes were detected by western blotting. In C2C12 cells overexpressing *PKM1* without SDSS treatment, the expression of *MyHC1* and *MyHC2a* decreased, while the expression of *MyHC2b* increased. However, the expression of *MyHCs* had no change in cells overexpressing PKM1 and treated with SDSS (Figures 6B, C). Those results show SDSS modulates *MyHCs* expression and muscle fiber type formation by blocking PKM1 activity.

## 4 Discussion

Muscle fiber type is an important factor influencing muscle metabolism and function as well as meat quality traits. Several natural compounds have been shown to promote muscle proliferation and differentiation (Ahmad et al., 2024) and to induce the transformation of glycolytic muscle fibers into oxidative muscle fibers (Jiang et al., 2019; Wen et al., 2020). Although SDSS has various pharmacological effects (MEIm et al., 2019), its effects on the formation of skeletal muscle fibers and muscle metabolism have not been examined. In this study, C57BL/6 mice were orally administered SDSS by gavage. The proportion of slow-twitch muscle fibers of SDSS treated mice were significantly higher than those of control mice. An increase in oxidative muscle fiber proportion leads to an increase muscle endurance and SDH activity (Jia et al., 2015; Zierath and Hawley, 2004). Composition of muscle fiber types is significantly associated with meat quality of agricultural animals (Lefaucheur, 2010; Ryu and Kim, 2005). SDSS treated mice have a higher proportion of slow-twitch muscle fibers and a larger muscle fiber cross-sectional area than control group mice, which may affect Meat quality traits, such as meat color, post slaughter pH value and drip loss, and further research are needed to verify in pigs. Muscle fiber type affects whole-body physiology and metabolism (Meng et al., 2013). In humans, glucose tolerance and insulin sensitivity are closely related to muscle fiber type, and both are affected by an increase in the number of oxidative muscle fibers (Snow and Thompson, 2009). This relationship is explained by the higher oxygen consumption and higher metabolic capacity of oxidative muscle fibers than glycolytic muscle fibers, which is consistent with previous study (Motohashi et al., 2009; Pataky et al., 2017).

Skeletal muscle fiber-type remodeling involves several key proteins and signaling pathways. In our study, we found SDSS target proteins Pyruvate kinase M (PKM) through DARTS and MS analysis. PKM1 was protected in the SDSS-treated group whereas PKM2 was degraded by thermolysin. The difference might have been due to differences in amino acid binding by SDSS, but this needs to be further studied. Phenylalanine is an allosteric inhibitor of PKM1 that causes a decrease in the enzyme’s apparent affinity for P-enolpyruvate (PEP) (Carminatti et al., 1971). The allosteric binding site for phenylalanine on PKM1 had been identified based on the cocrystal structure of PKM1 with Ala bound (PDB code: 2G50) and was a deep pocket between the A and C domains and distant from the active site. His463, Arg105, and Ile468 are key residues in the interaction with phenylalanine (Williams et al., 2006). In view of the structural similarity of SDSS and phenylalanine, PKM1 inhibition by the former is likely mediated by allosteric interactions. Accordingly, SDSS might successfully insert into the allosteric binding groove of PKM1, where it interacted with enzyme residues.

Pyruvate kinase M1/2 (PKM1/2) regulates the glycolytic system and is a rate-limiting glycolytic enzyme (Chen et al., 2010). PKM1 is predominantly expressed in high-energy (glucose)-demanding) organs such as brain and muscle. In muscle, the cross-sectional area of glycolytic fibers is higher than that of oxidative fibers (Maltin et al., 1998). In a previous study, PKM1 deletion led to a significant decrease in cardiomyocyte cross-sectional area (Li et al., 2021). PKM1 knockdown in C2C12 myoblasts increased the expression of *MyHC1* and *MyHC2a* and decreased that of *MyHC2b*, suggesting that PKM1 promotes the formation of glycolytic muscle fibers. We also found that SDSS significantly inhibited PK activity, although the rate of inhibition was not very high. The ability of SDSS to suppress the effect of PKM1 on the expression of genes involved in muscle fiber type determination indicates that the SDSS-mediated transformation from a glycolytic into an oxidative fiber type in skeletal muscle depends on PKM1 activity. PKM1 may act as a metabolic enzyme to regulate muscle metabolism and affect the conversion of muscle fiber types, or PKM1 may also act as a transcription factor that directly binds to gene promoters to regulate the expression of muscle fiber type related genes. The molecular mechanism by which PKM1 regulates MyHC gene expression needs further investigation.

## 5 Conclusion

In conclusion, our results show that SDSS promotes fast glycolytic-to-slow oxidative muscle fiber type transformation, and enhances the oxidative capacity of skeletal muscle *in vivo*. Mechanistically, we identified PKM1 as a potential binding protein of SDSS by DARTS, mass spectrometry and molecular docking analyses. SDSS bound to the pyruvate kinase M1 and significantly repressed its activity. Additionally, PKM1 inhibited MyHC1 and MyHC2a expression but promoted MyHC2b expression. SDSS significantly attenuated the effects of PKM1 on muscle fiber-related gene expression in C2C12 myoblasts.

## Data Availability

The datasets presented in this study can be found in online repositories. The names of the repository/repositories and accession number(s) can be found in the article/Supplementary Material.
