# Supplementary material for: Sodium danshensu modulates skeletal muscle fiber type formation and metabolism by inhibiting pyruvate kinase M1
Source: Front Pharmacol. 2024 Oct 22;15:1467620. doi: 10.3389/fphar.2024.1467620 (PMC11534700; doi:10.3389/fphar.2024.1467620)
Supplement: Supplementary file 1 [file DataSheet2.pdf]

Figure 1B

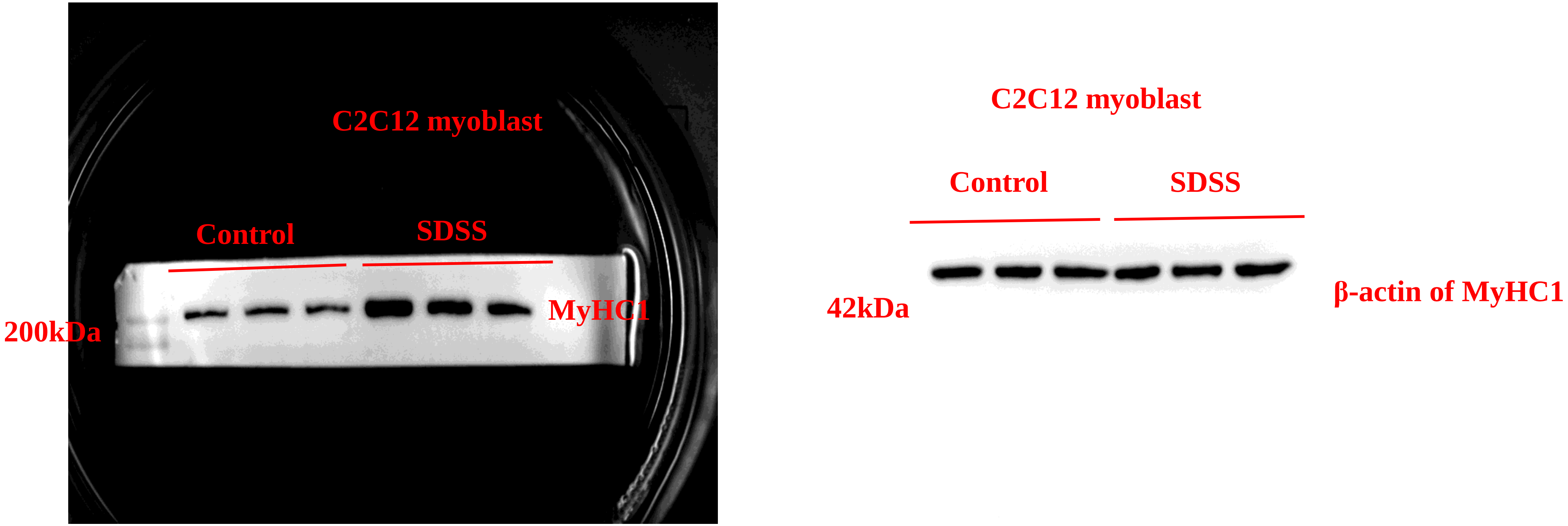
C2C12 myoblast
Control
SDSS
MyHC1
200kDa
C2C12 myoblast
Control
SDSS
42kDa
β-actin of MyHC1

Figure 1B

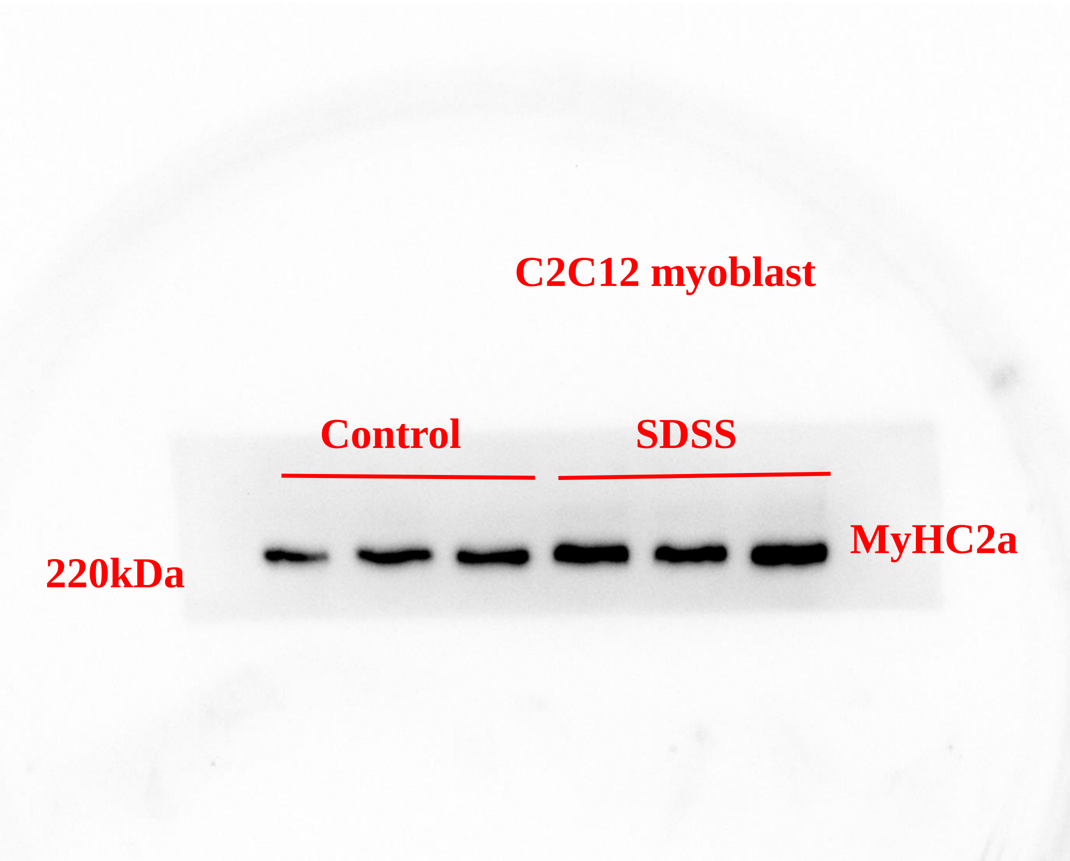
C2C12 myoblast
Control
SDSS
220kDa
MyHC2a

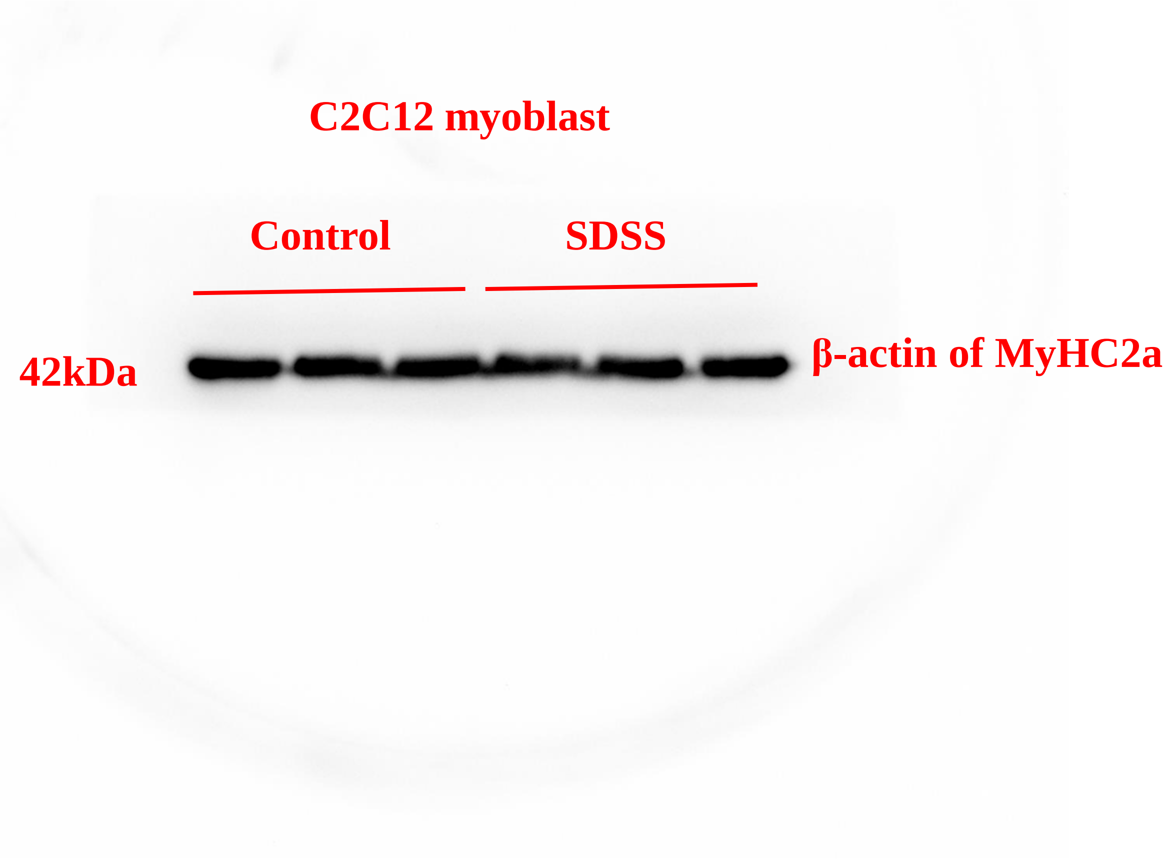
C2C12 myoblast
Control
SDSS
42kDa
β-actin of MyHC2a

Figure 1B

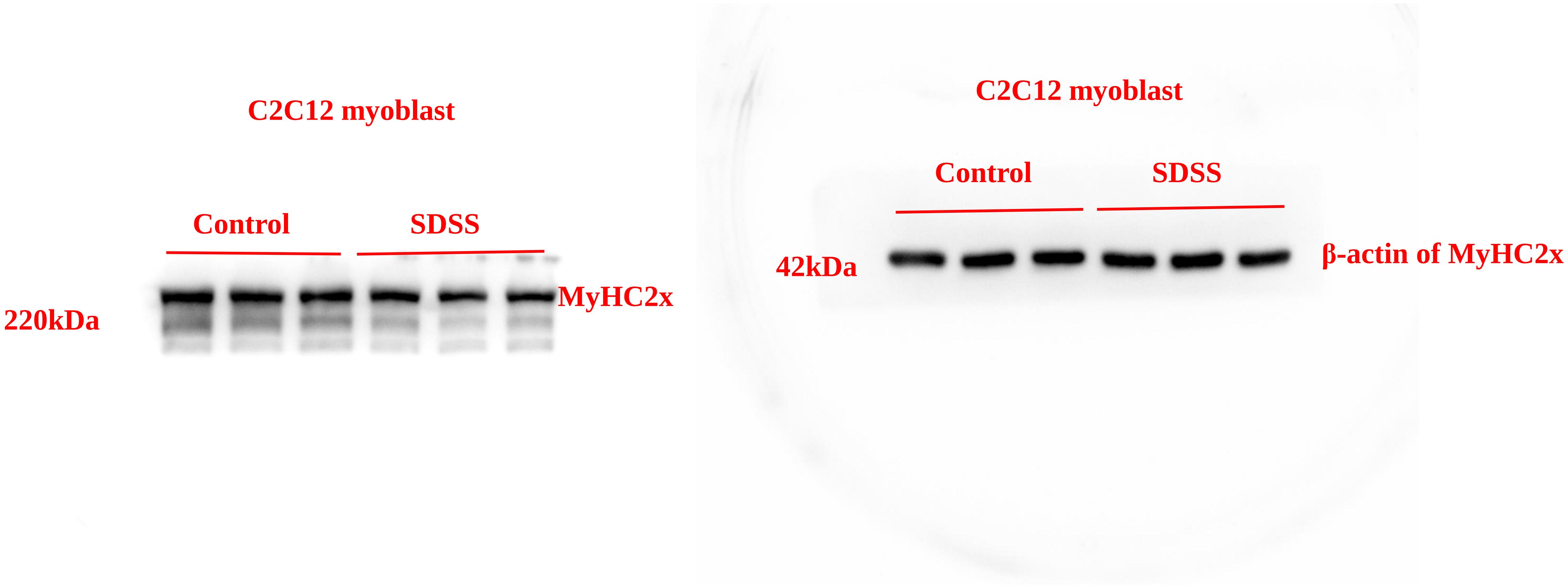
C2C12 myoblast
Control
SDSS
220kDa
MyHC2x
C2C12 myoblast
Control
SDSS
42kDa
β-actin of MyHC2x

Figure 1B

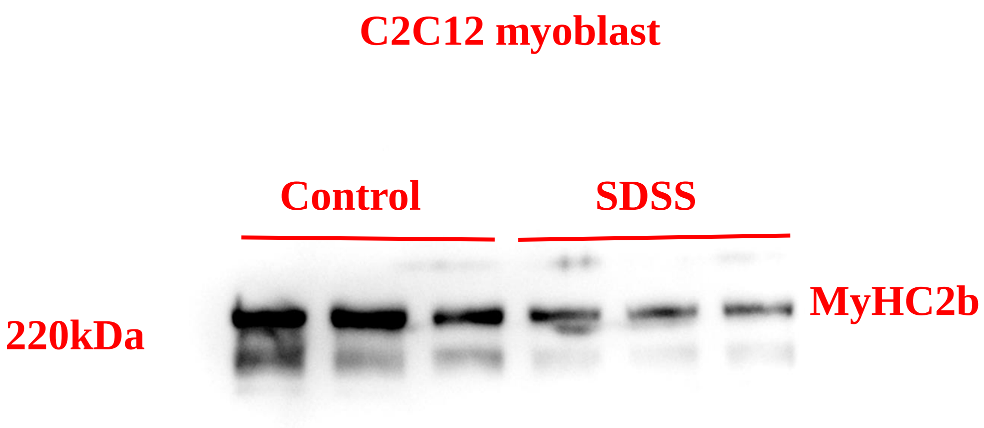
C2C12 myoblast
Control
SDSS
220kDa
MyHC2b

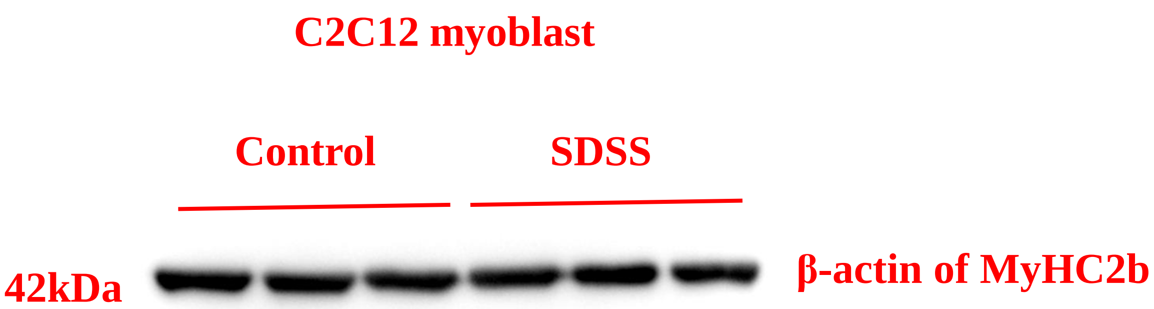
C2C12 myoblast
Control
SDSS
42kDa
β-actin of MyHC2b

Figure 2C

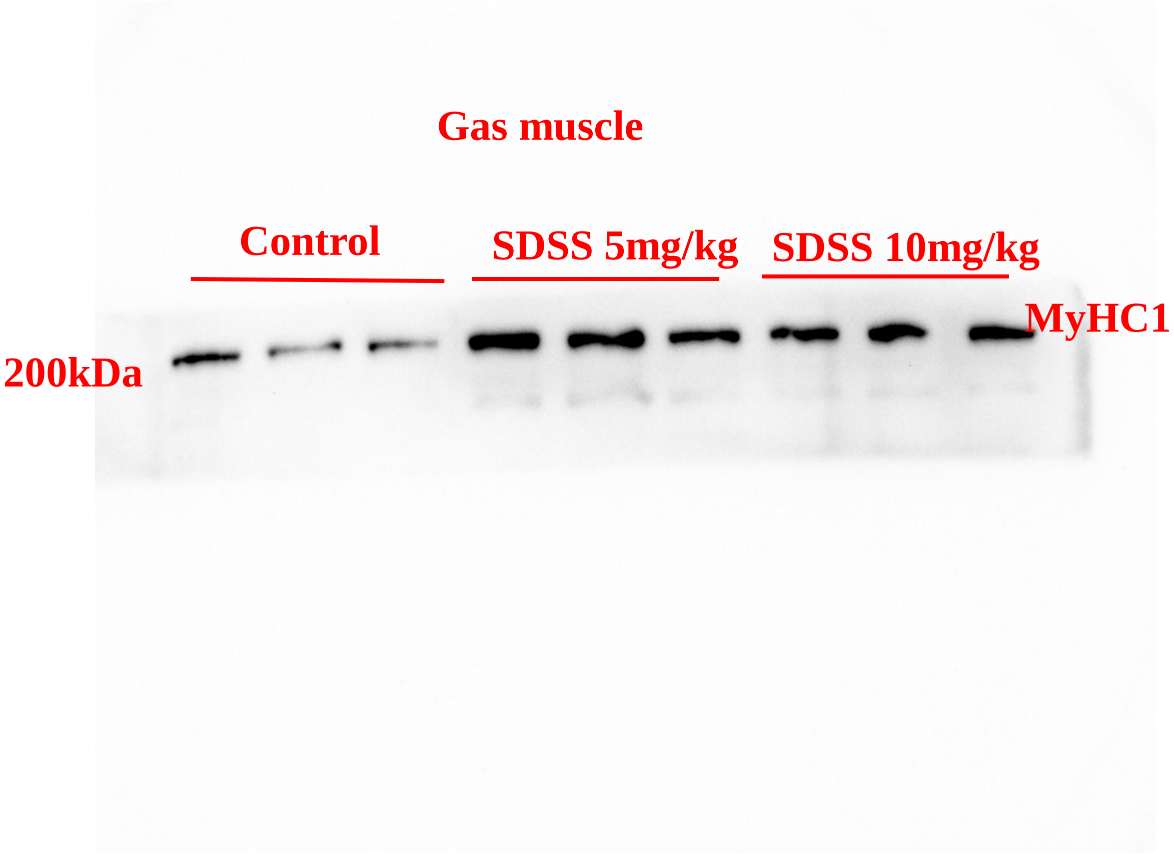

Gas muscle
Control
SDSS 5mg/kg
SDSS 10mg/kg
MyHC1
200kDa

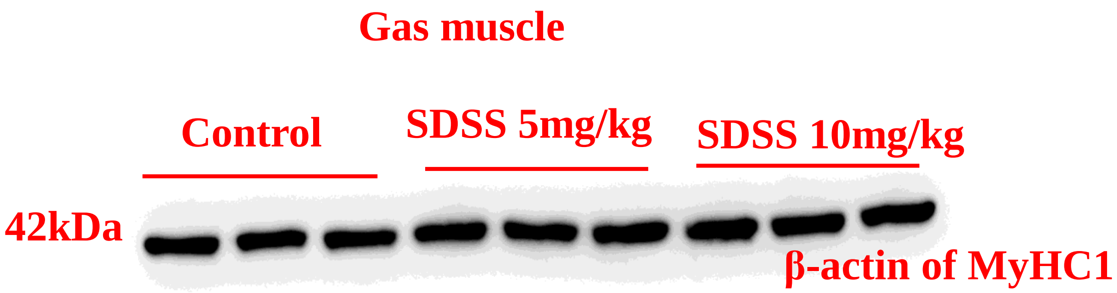

Gas muscle
Control
SDSS 5mg/kg
SDSS 10mg/kg
42kDa
β-actin of MyHC1

Figure 2C

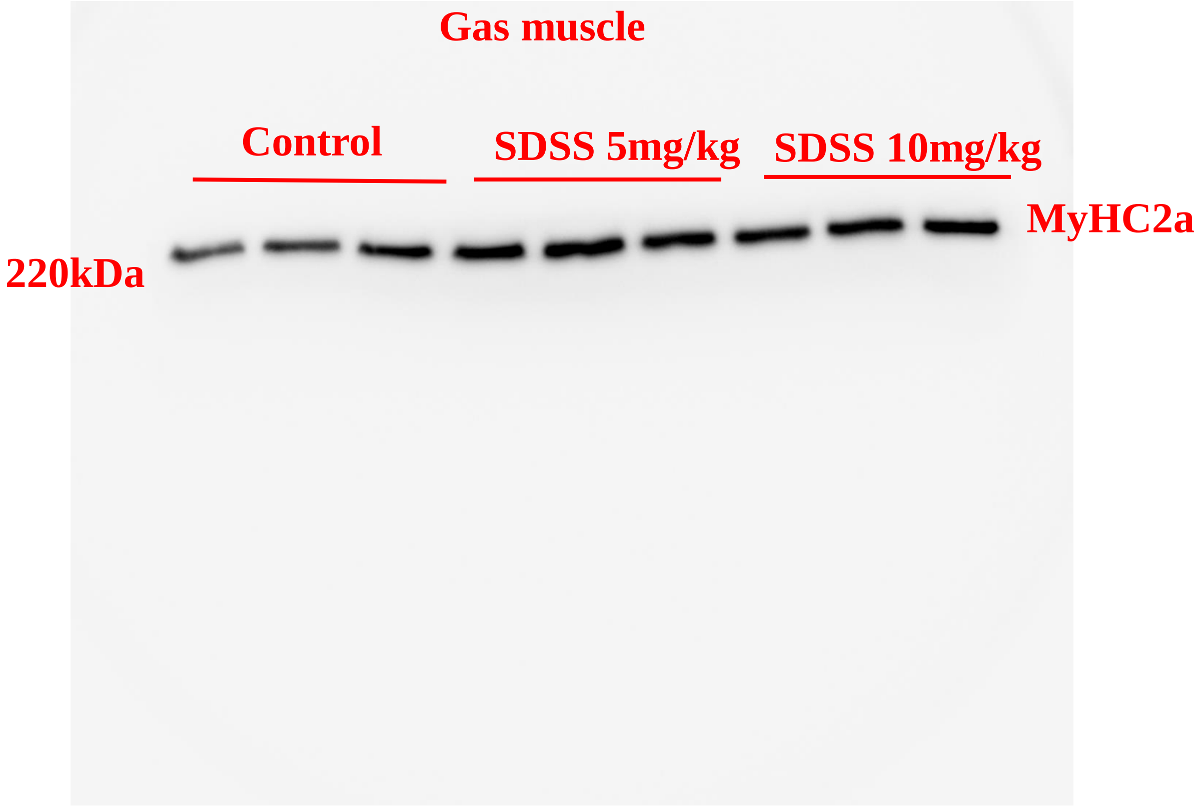
Gas muscle
Control
SDSS 5mg/kg
SDSS 10mg/kg
MyHC2a
220kDa

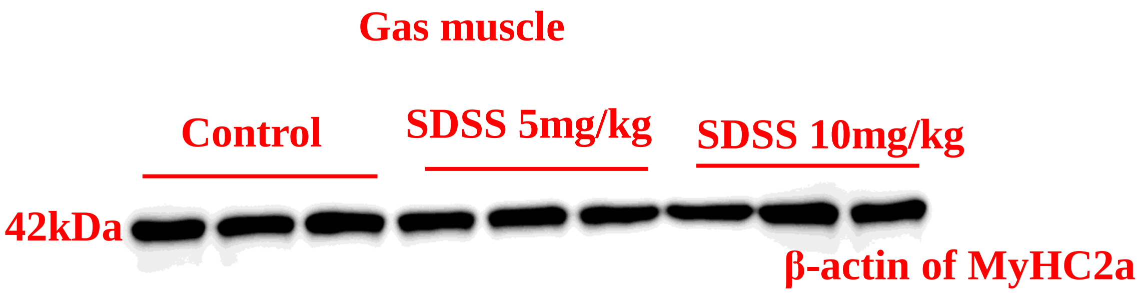
Gas muscle
Control
SDSS 5mg/kg
SDSS 10mg/kg
42kDa
β-actin of MyHC2a

Figure 2C

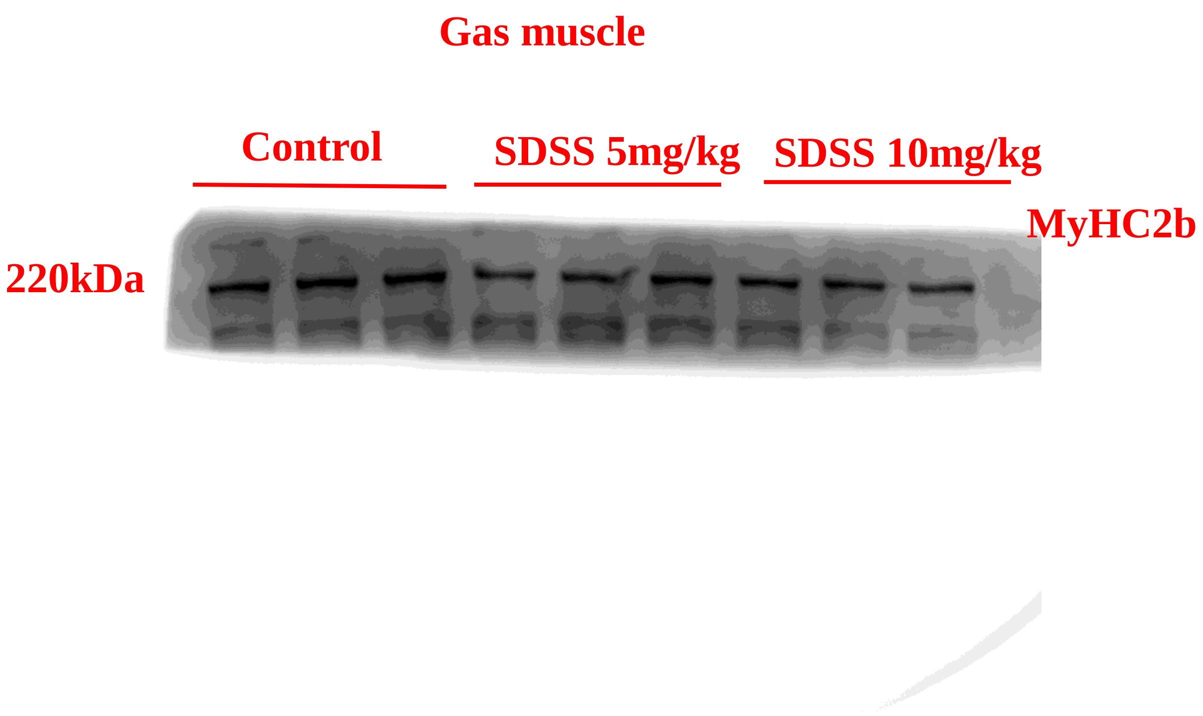
Gas muscle
Control
SDSS 5mg/kg
SDSS 10mg/kg
220kDa
MyHC2b

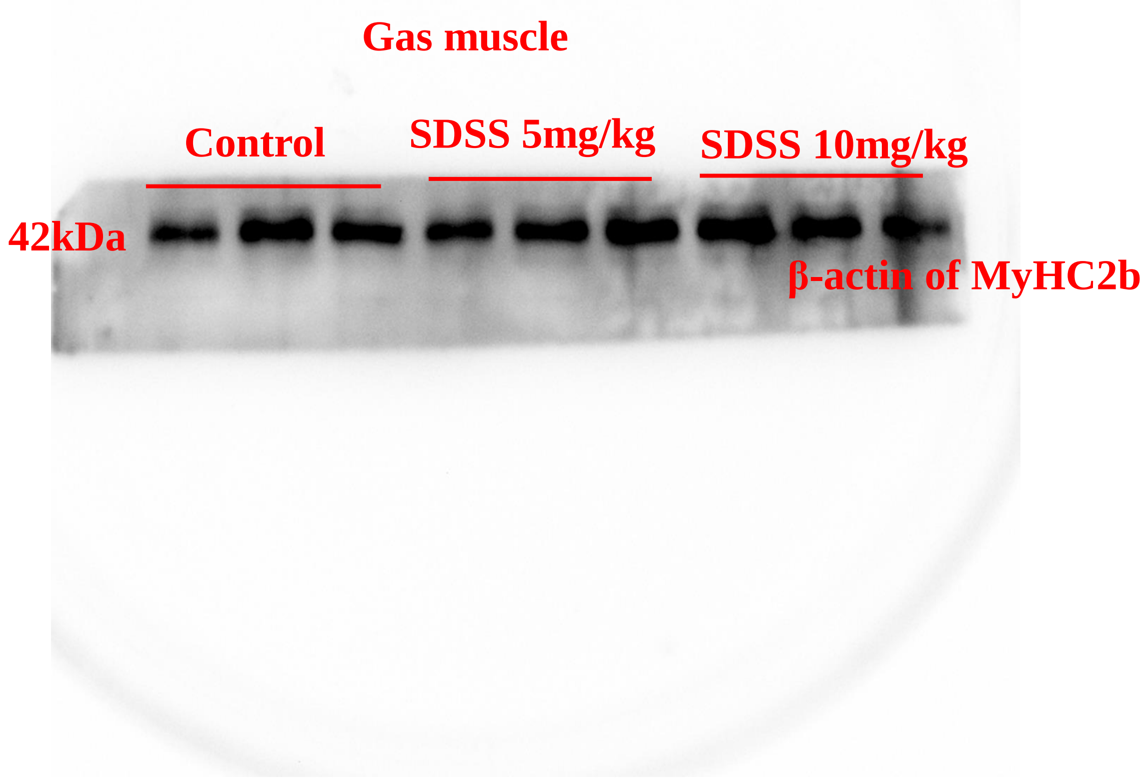
Gas muscle
Control
SDSS 5mg/kg
SDSS 10mg/kg
42kDa
β-actin of MyHC2b

Figure 5D

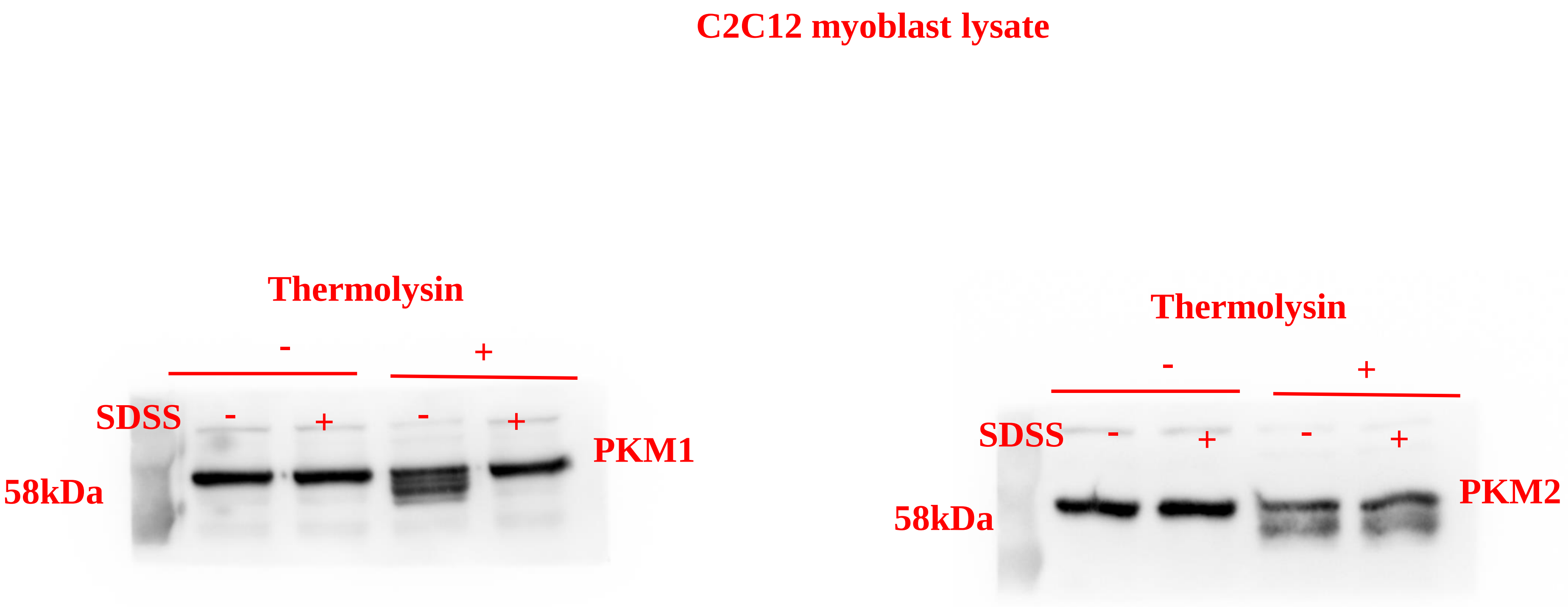
C2C12 myoblast lysate
Thermolysin
-
+
SDSS
-
+
-
+
PKM1
58kDa
Thermolysin
-
+
SDSS
-
+
-
+
PKM2
58kDa

Figure 5F

C2C12 myoblast

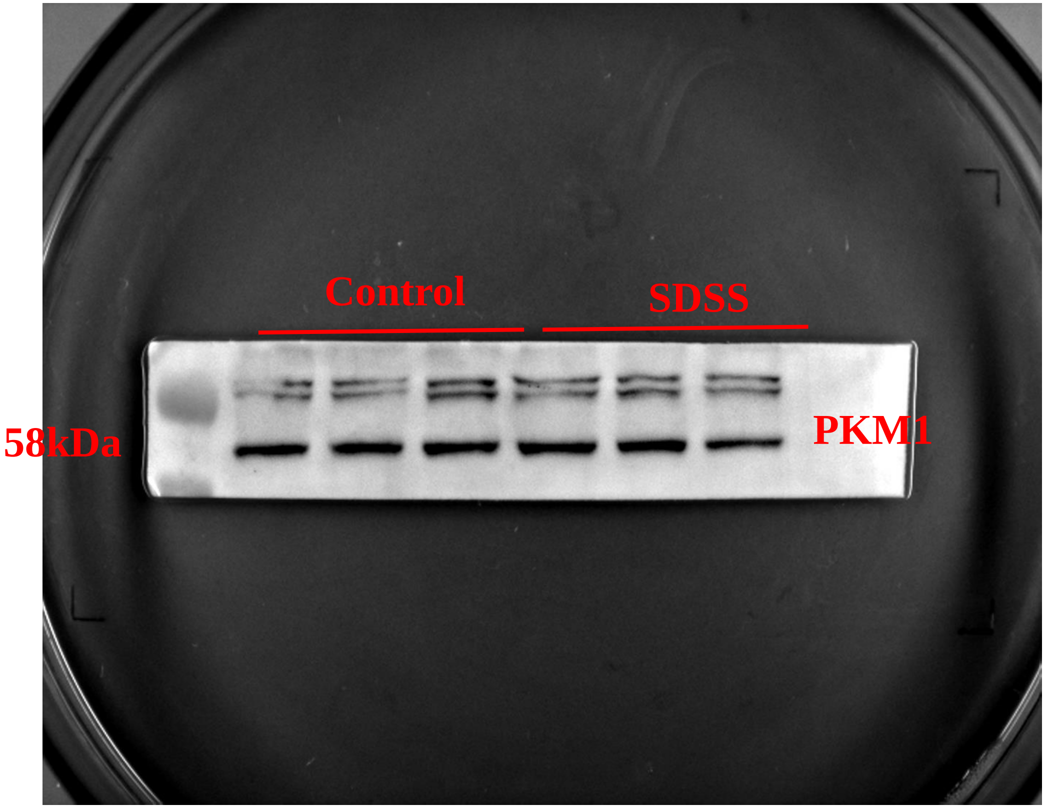

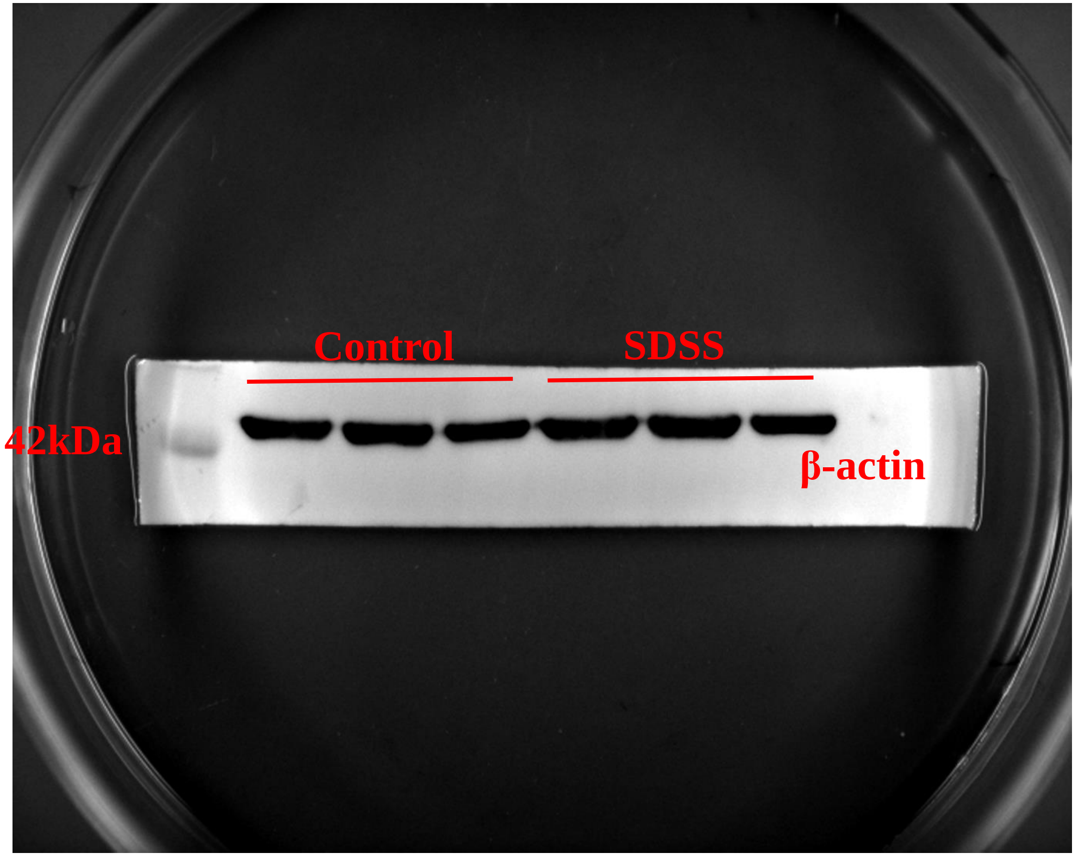

Figure 5I

C2C12 myoblast

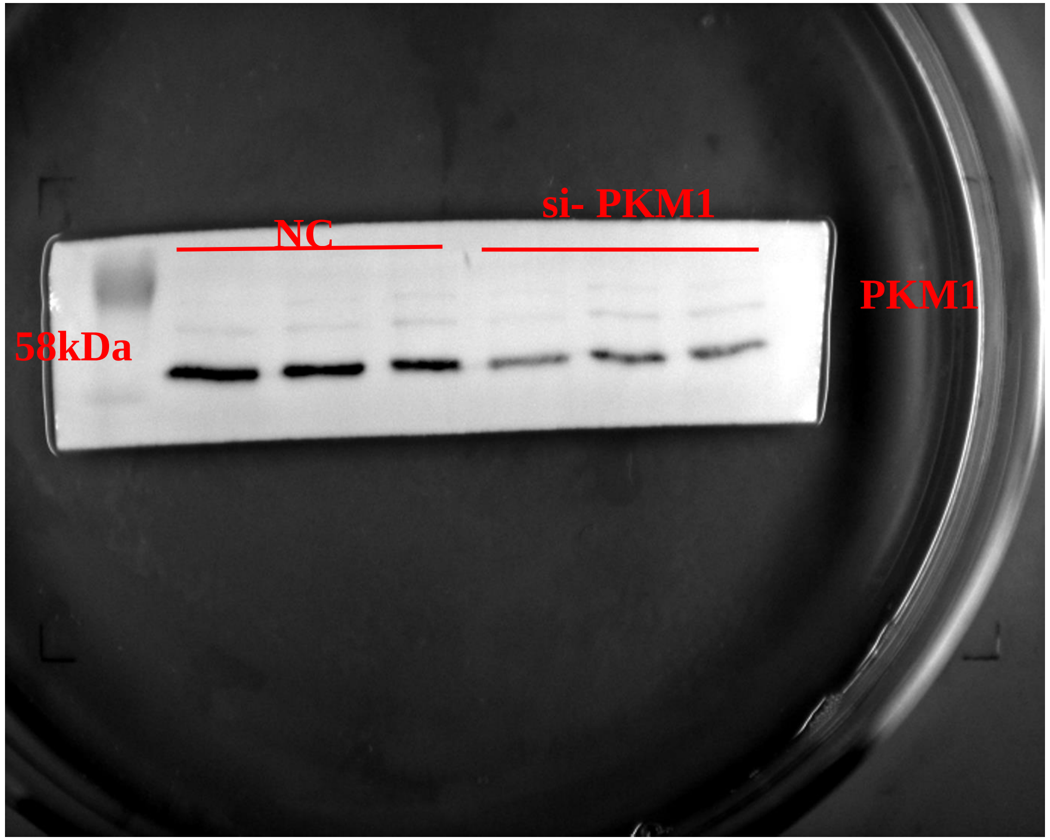

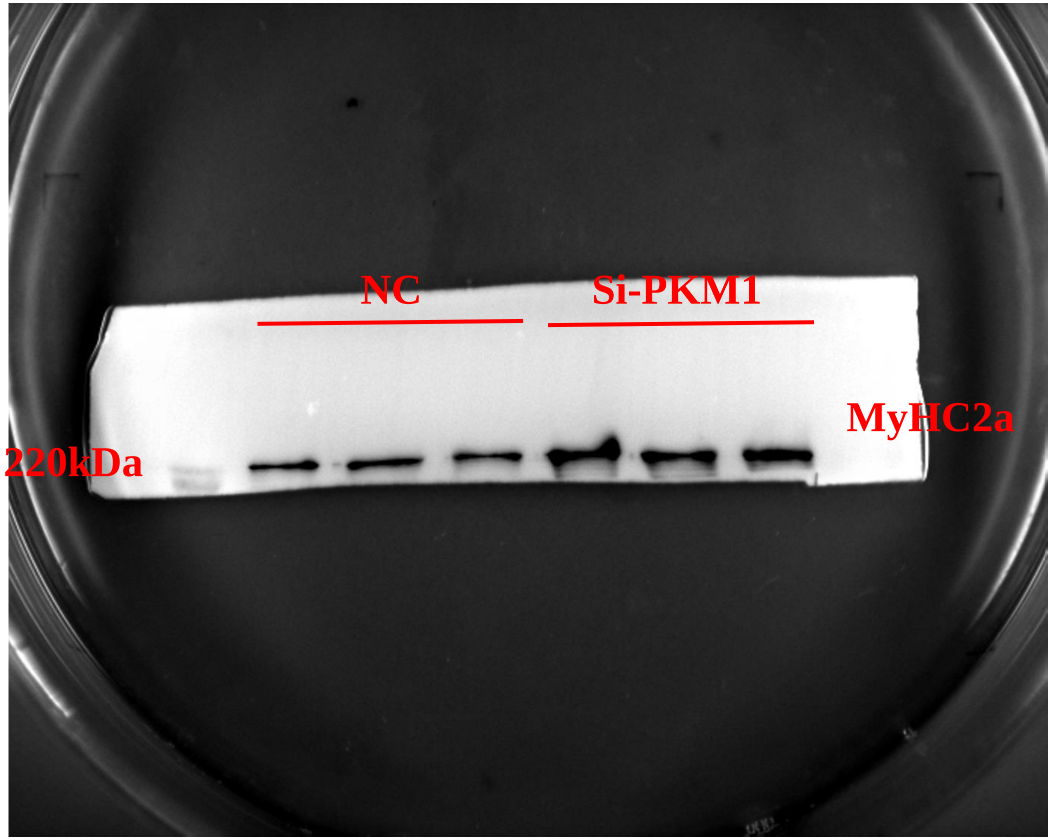

Figure 5I

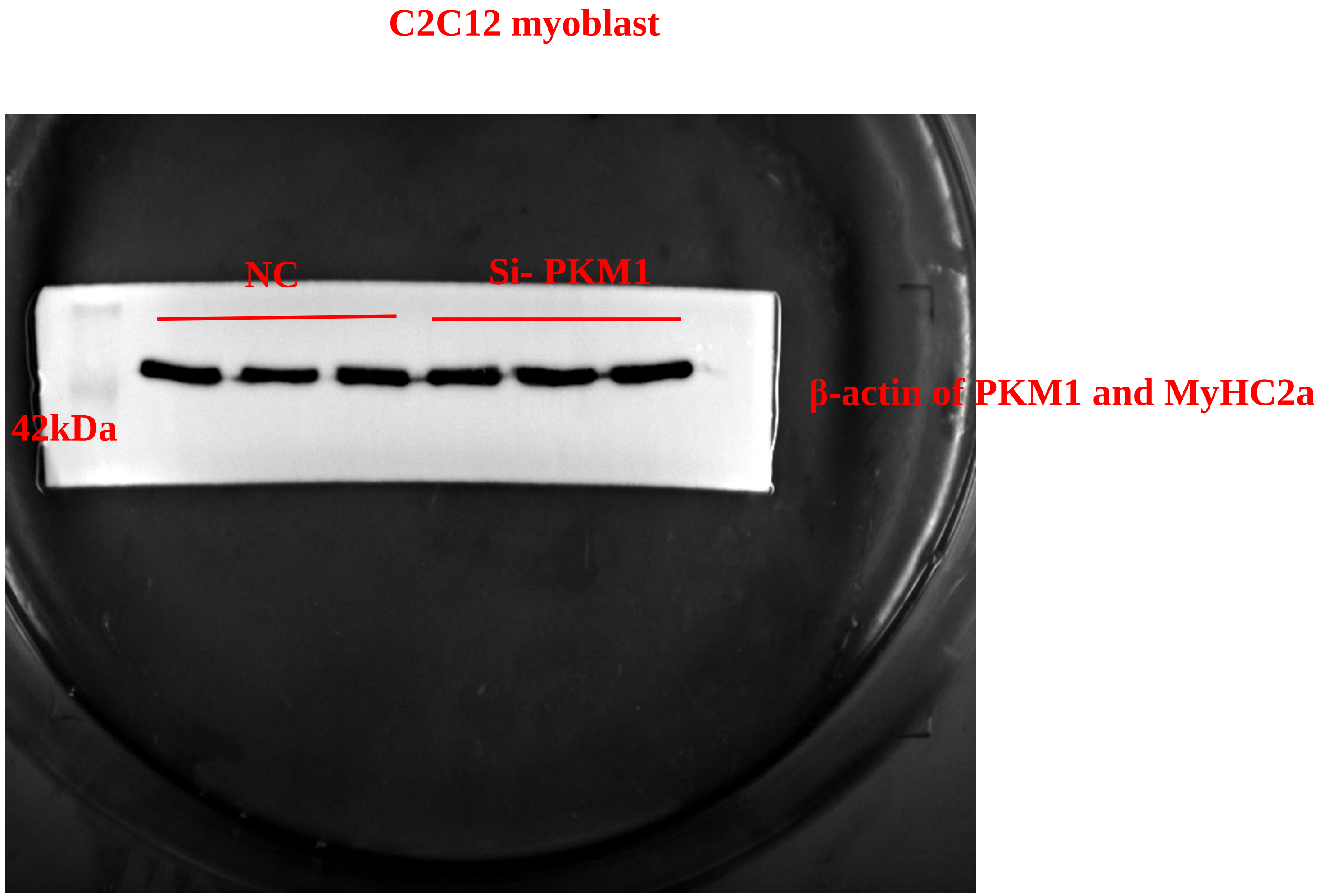

Figure 5I

C2C12 myoblast

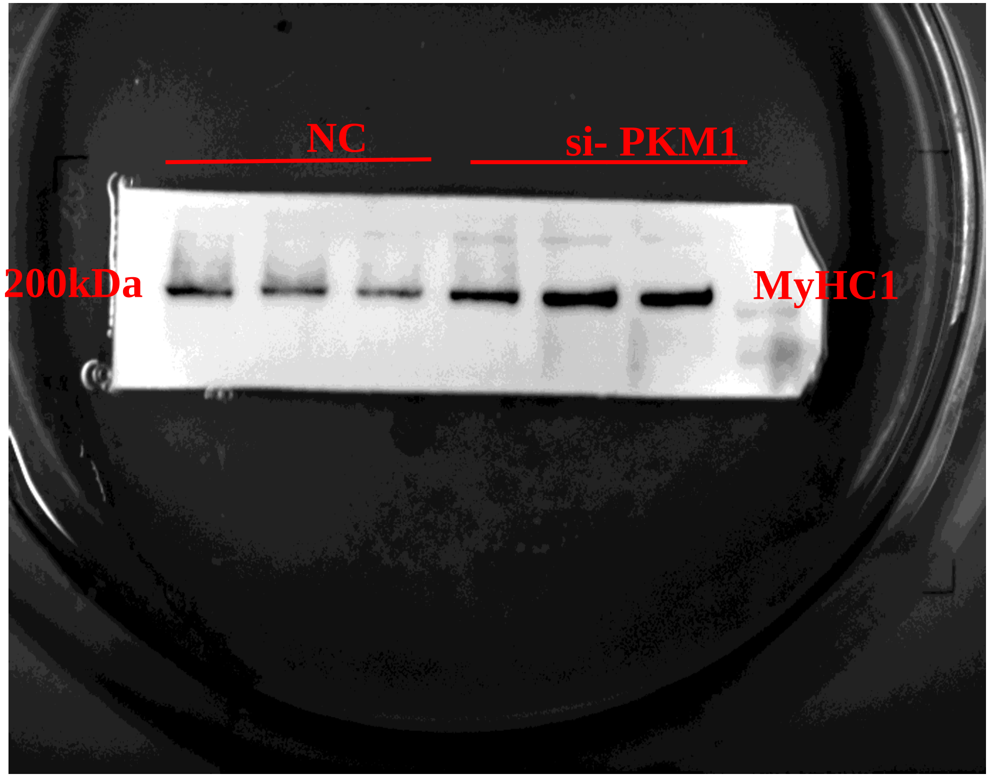

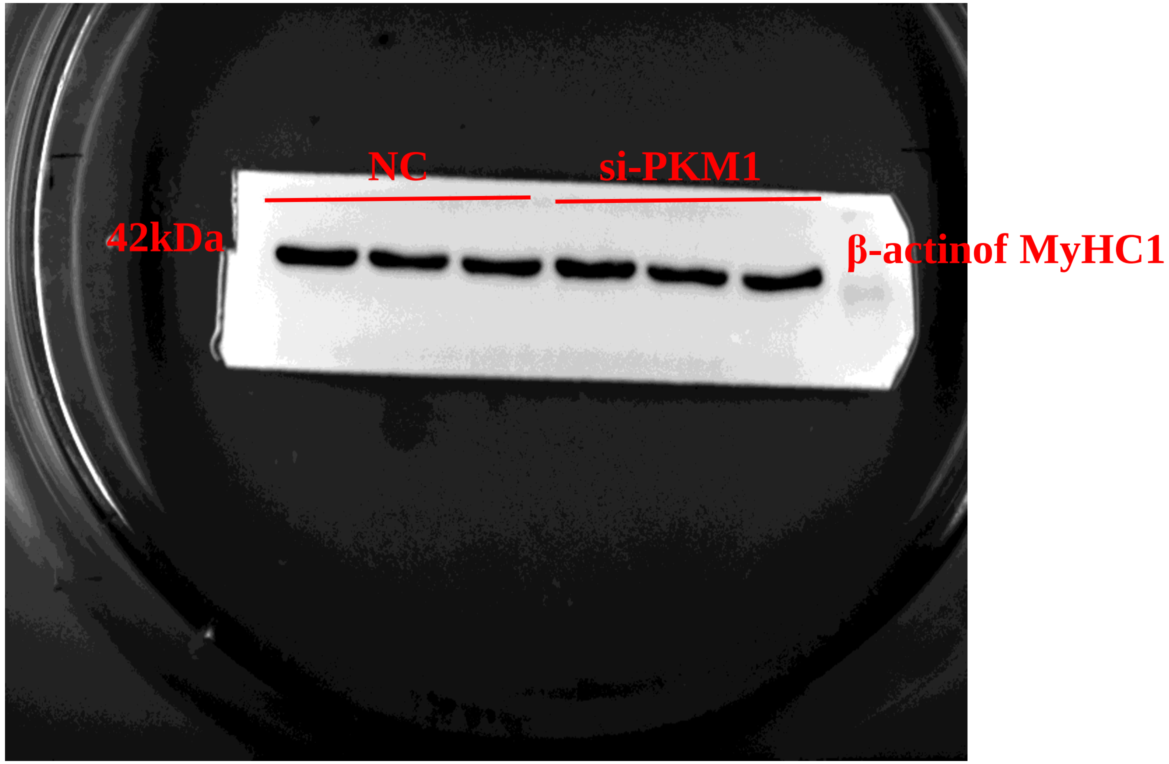

Figure 5I

C2C12 myoblast

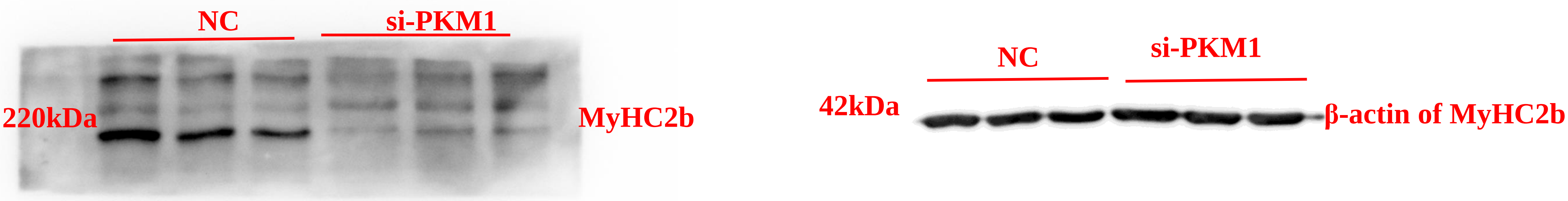

Figure 6B

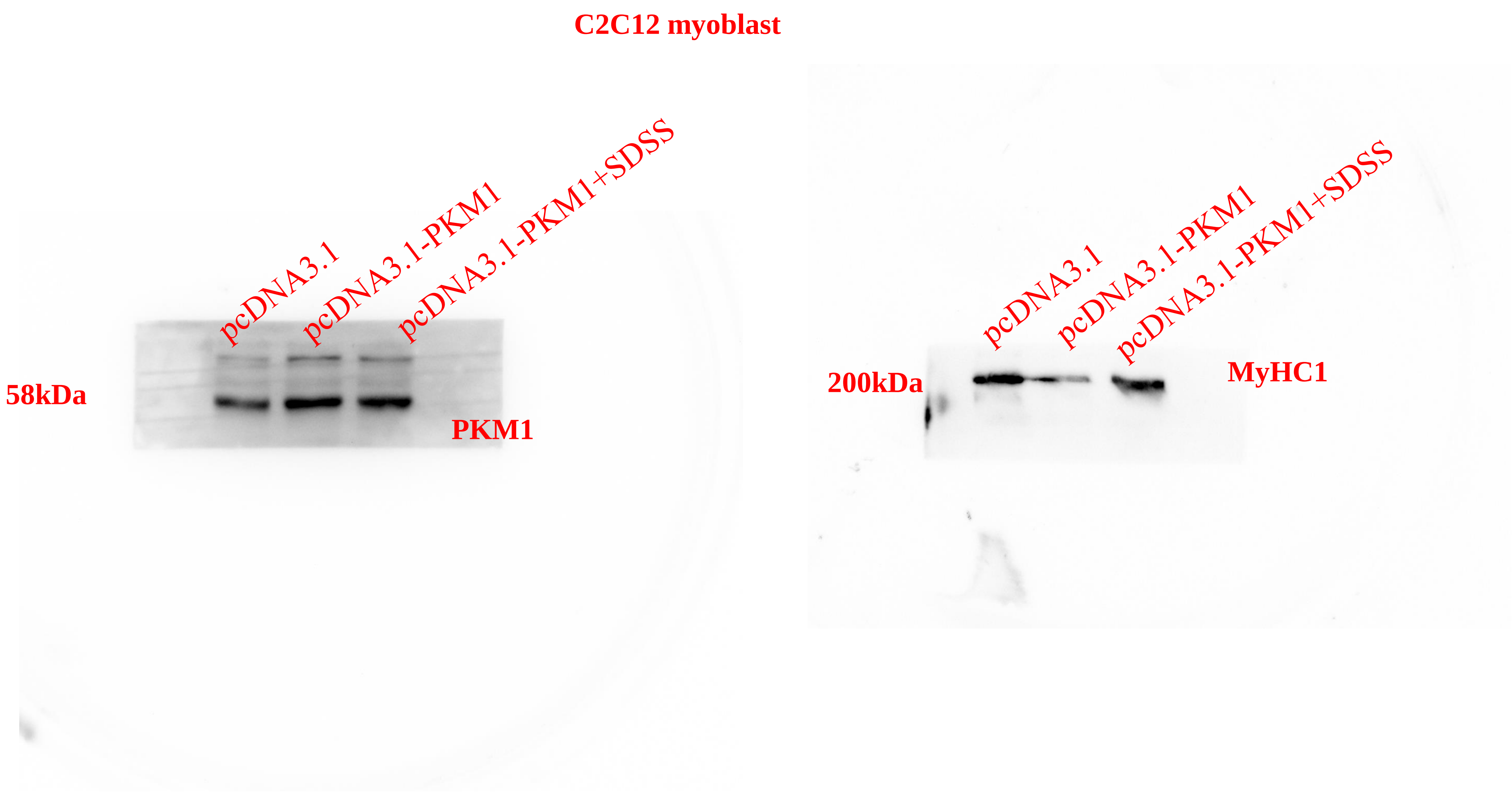
C2C12 myoblast
pcDNA3.1
pcDNA3.1-PKM1
pcDNA3.1-PKM1+SDSS
58kDa
PKM1
pcDNA3.1
pcDNA3.1-PKM1
pcDNA3.1-PKM1+SDSS
200kDa
MyHC1

Figure 6B

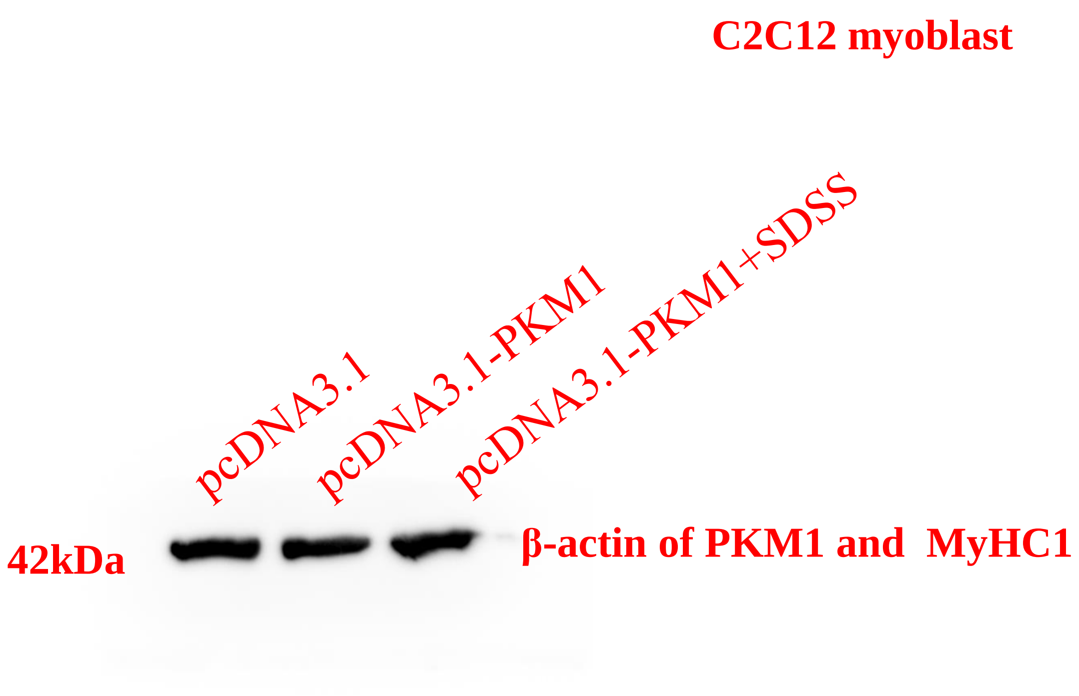
C2C12 myoblast
pcDNA3.1
pcDNA3.1-PKM1
pcDNA3.1-PKM1+SDSS
42kDa
β-actin of PKM1 and MyHC1

Figure 6B

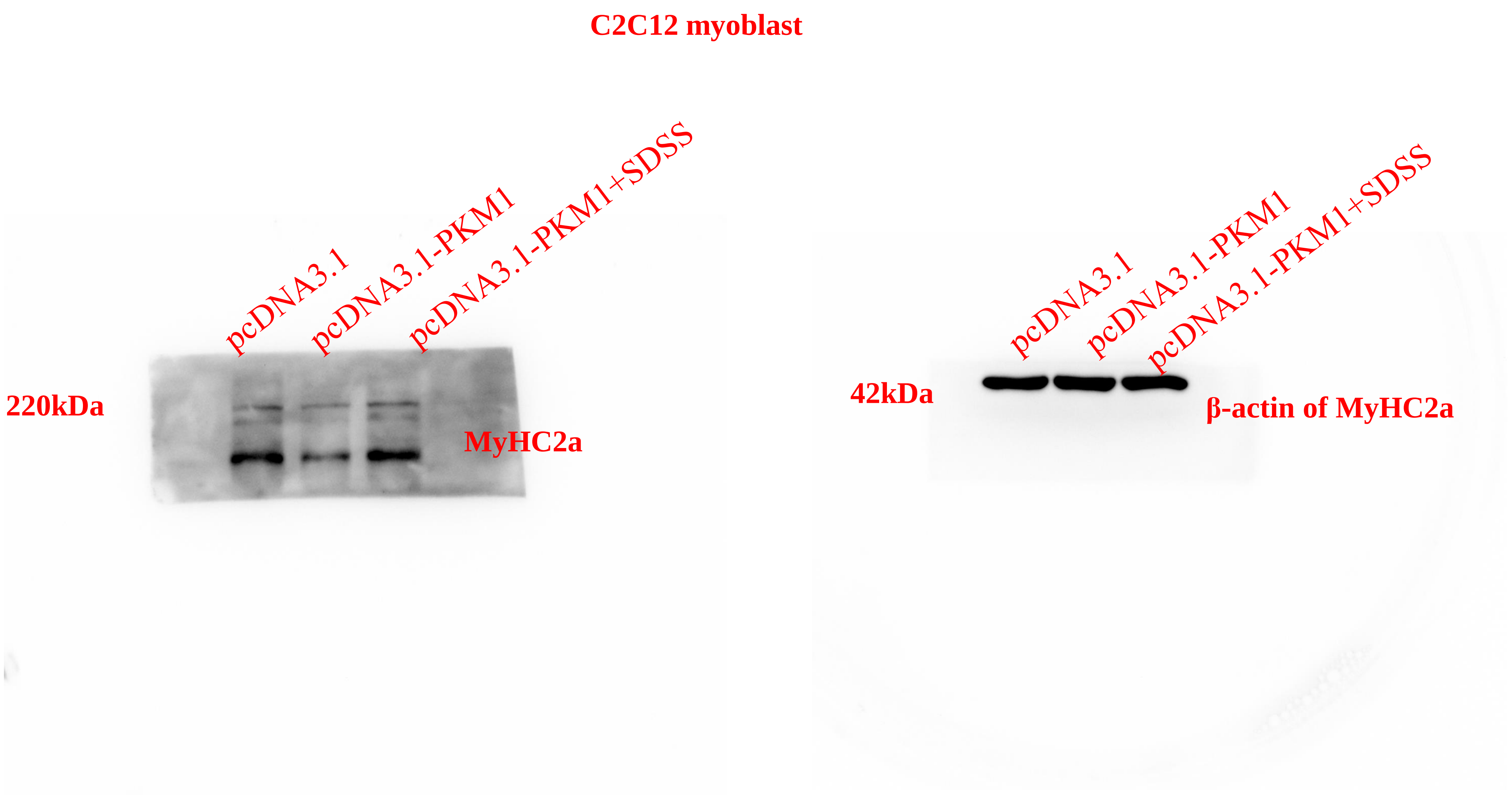
C2C12 myoblast
pcDNA3.1
pcDNA3.1-PKM1
pcDNA3.1-PKM1+SDSS
220kDa
MyHC2a
pcDNA3.1
pcDNA3.1-PKM1
pcDNA3.1-PKM1+SDSS
42kDa
β-actin of MyHC2a

Figure 6B

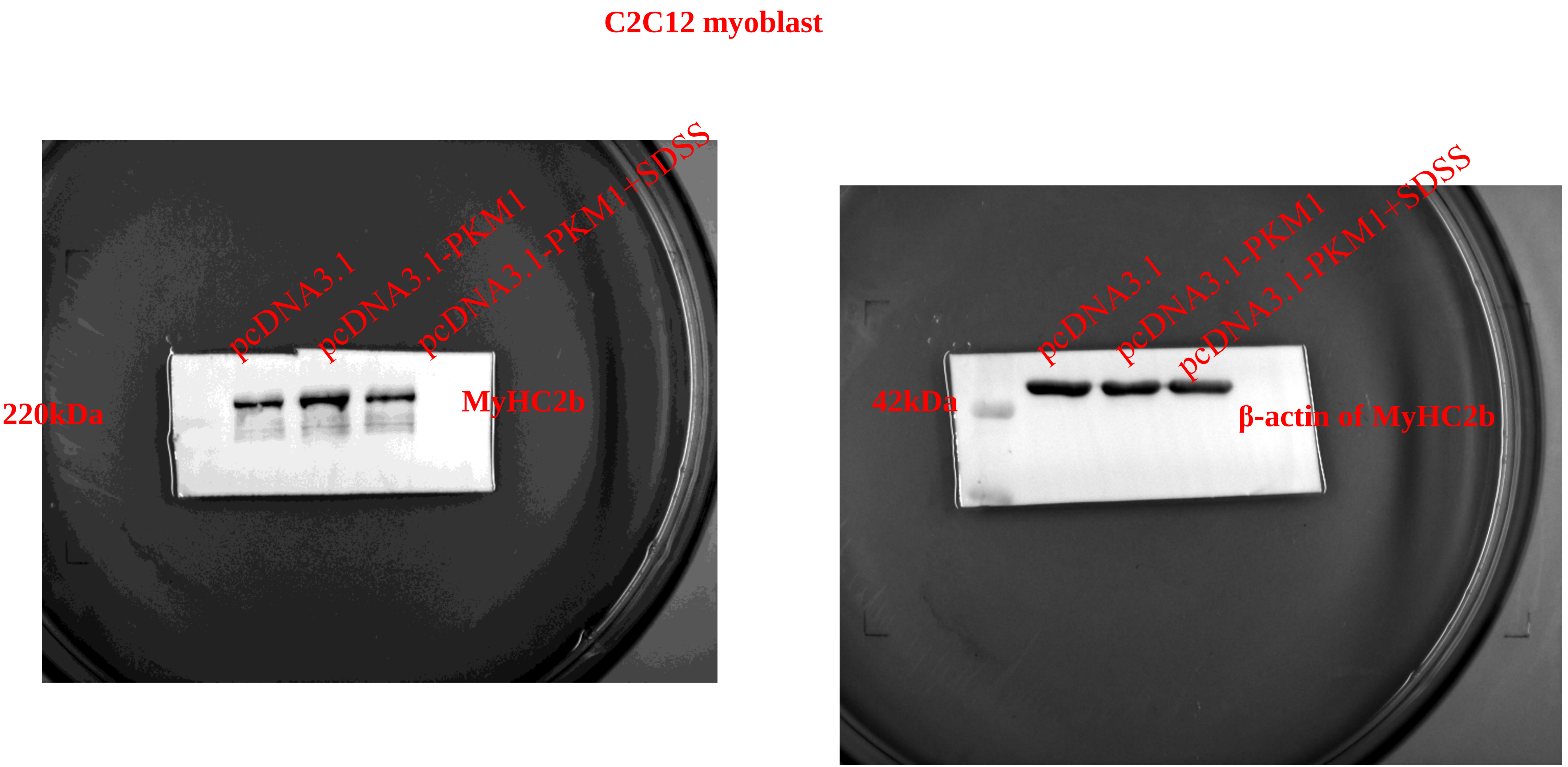
C2C12 myoblast
pcDNA3.1
pcDNA3.1-PKM1
pcDNA3.1-PKM1+SDSS
220kDa
MyHC2b
pcDNA3.1
pcDNA3.1-PKM1
pcDNA3.1-PKM1+SDSS
42kDa
β-actin of MyHC2b

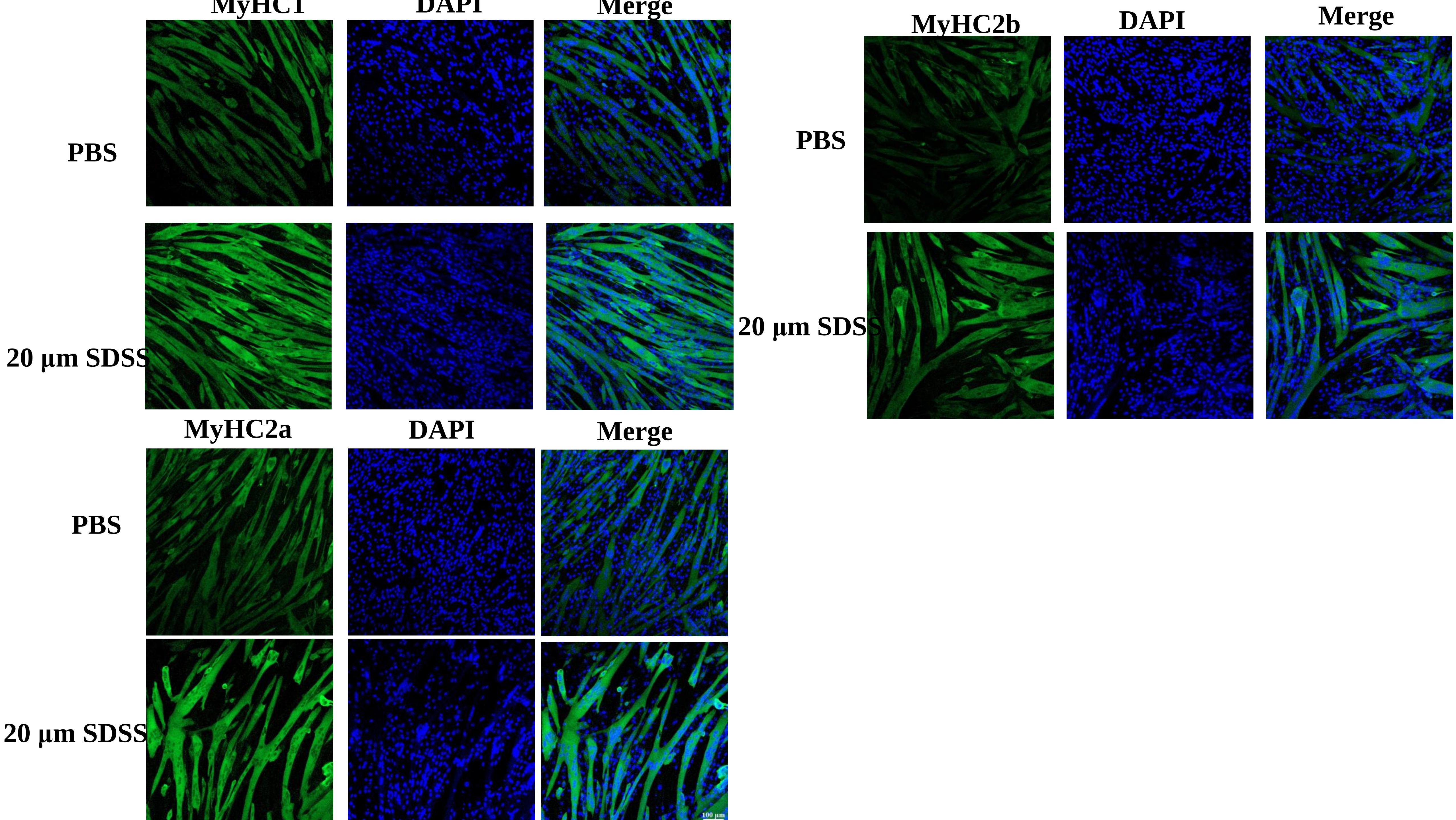
MyHC1
DAPI
Merge
PBS
20 μm SDSS
MyHC2a
DAPI
Merge
PBS
20 μm SDSS
MyHC2b
DAPI
Merge
PBS
20 μm SDSS
100 μm

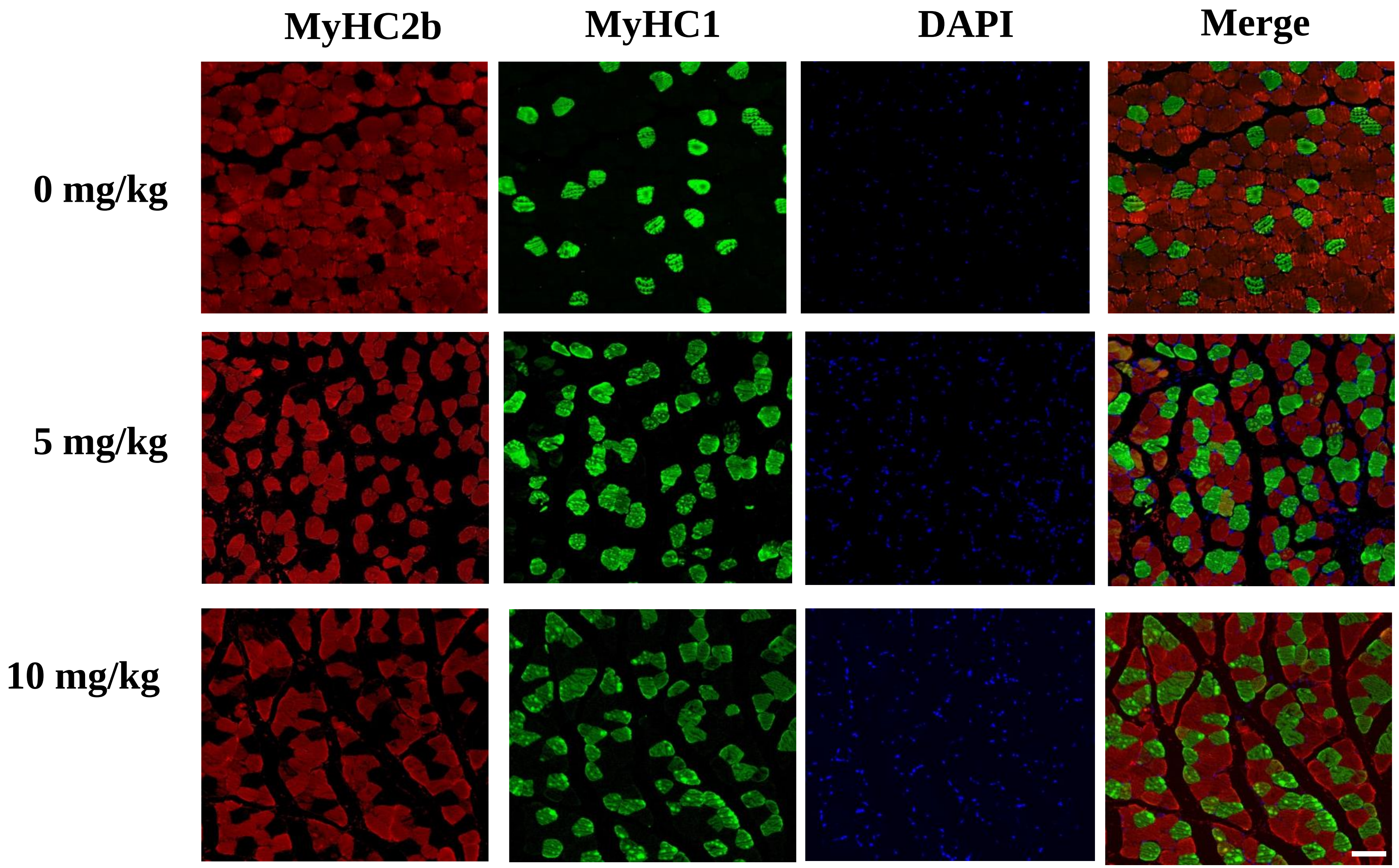
MyHC2b
MyHC1
DAPI
Merge
0 mg/kg
5 mg/kg
10 mg/kg
