# Supplementary material for: Sodium danshensu modulates skeletal muscle fiber type formation and metabolism by inhibiting pyruvate kinase M1
Source: Front Pharmacol. 2024 Oct 22;15:1467620. doi: 10.3389/fphar.2024.1467620 (PMC11534700; doi:10.3389/fphar.2024.1467620)
Supplement: Supplementary file 3 [file DataSheet1.docx]

Supplementary Material

# Supplementary Material

**Cell viability assay**

C2C12 myoblasts were placed on a 96-well cell (104 cell per well). When the cells had attached, the original medium was removed and fresh medium with SDSS (0, 10, 20, 40, 60, 80, 100, 120 µM) was added to the plate. After 24 h incubation, the medium was removed and fresh medium with cell counting kit-8 solution (Solarbio, Beijing, China, CA1210) was added. After 1 h incubation, the absorbance at 450 nm was determined by a microplate reader (Bio Tek, United States, Synergy-HT). The cell viability of a well was the absorbance of the well with cell and cell culture medium subtracted from the well with cell culture medium only. The cell viability in the control (PBS (0 um)) group was normalized to 1. The relative viability (%)= [A (SDSS)－A (0)]/[ A (PBS)－A (0)]×100, A (0) is the absorbance of the well with cell culture medium only.

# Supplementary Figures
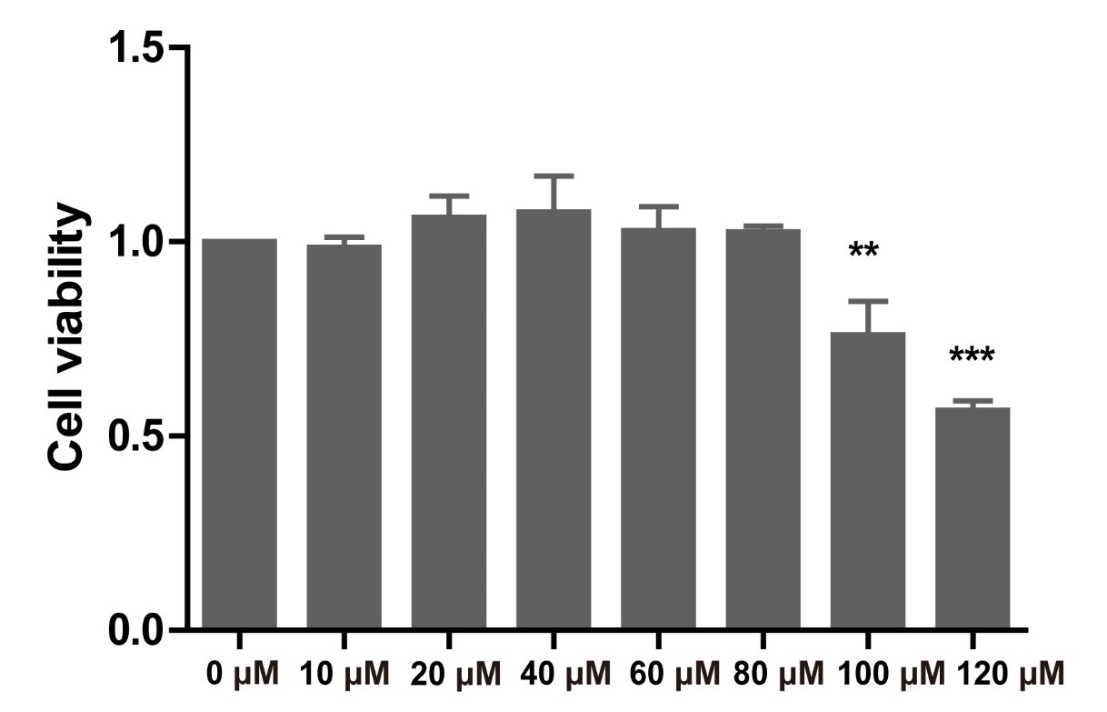


**Figure S1** The viability assay in C1C12 myoblasts. C2C12 treated for 24 h with 0, 10, 20, 40, 60, 80, 100, 120 μM SDSS (n=3). p < 0.01, p < 0.0001.


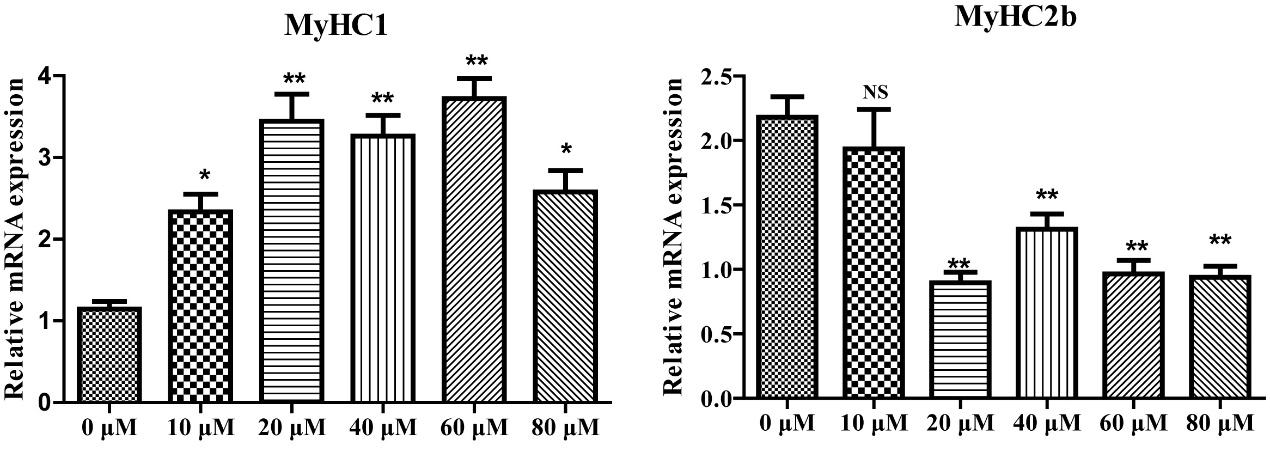


**Figure S2** Determination of the optimum concentration of SDSS. 80–90% confluent C2C12 myoblasts were cultured in the differentiation medium (DMEM, 2% horse serum) and treated with 0, 10, 20, 40, 60, and 80 μM SDSS for 4 days. qPCR analysis of mRNA expression of muscle fiber type-related genes. Data are presented as means ± SD. *p < 0.05, **p < 0.01.


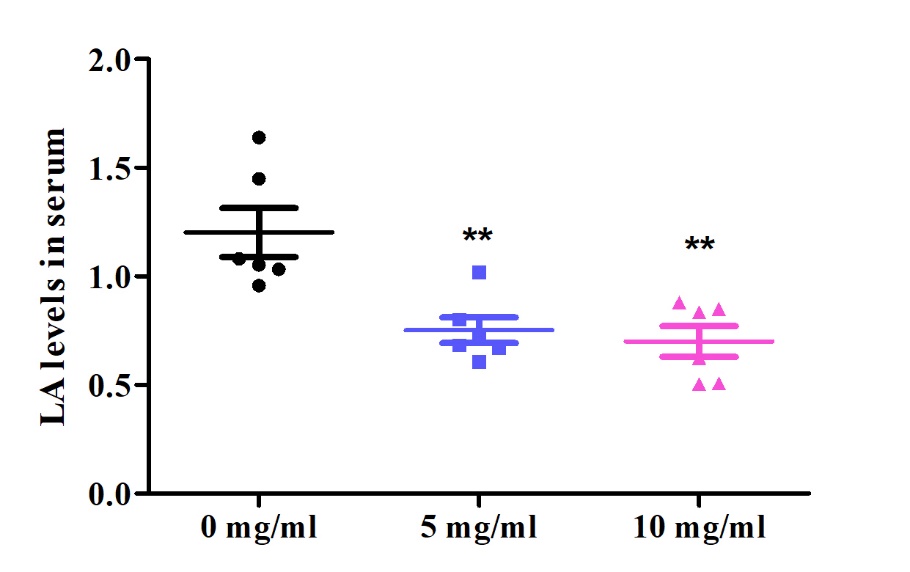


**Figure S3** The lactic acid levels in the serum of mice treated with SDSS orally once a day for 8 weeks (n = 6). **P < 0.01.


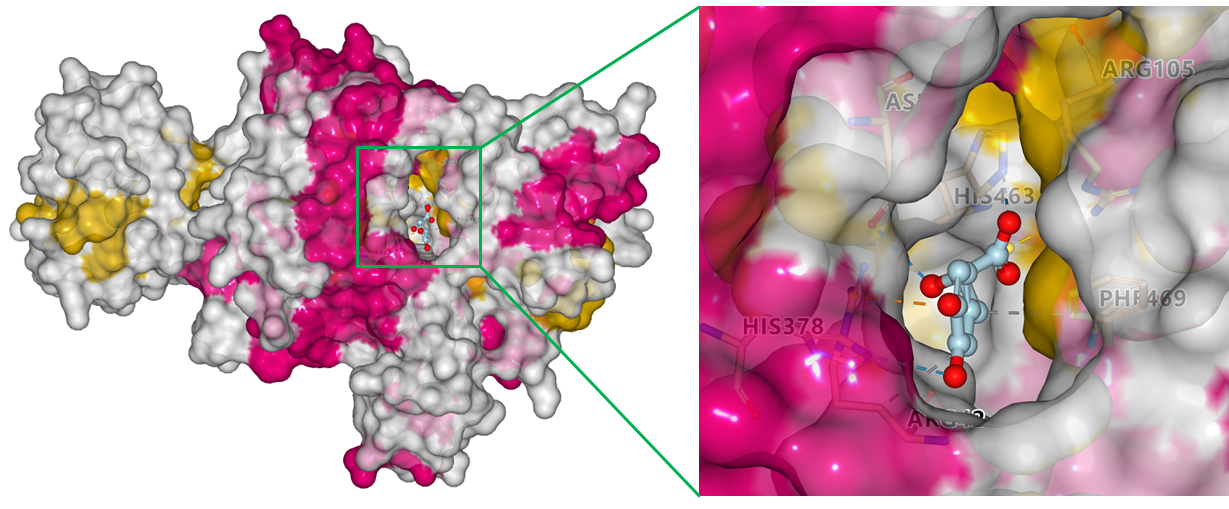


**Figure S4** Predicted dock conformation between SDSS and PKM1 (2G50).
